# Therapeutic effect of Yupingfeng powder and its bioactive metabolites on renal diseases

**DOI:** 10.3389/fphar.2026.1807950

**Published:** 2026-07-31

**Authors:** Yuxin Bai, Zhanhong Cao, Yue Shen, Chen Zhang, Xiaofan Yang

**Affiliations:** School of Chinese Medicine, Bozhou University, Bozhou, China

**Keywords:** bioactive metabolites, pharmacology, renal diseases, renoprotective mechanisms, Yupingfeng powder

## Abstract

Yupingfeng powder (YPFP), also known as Yupingfeng San, is a traditional Chinese medicine (TCM) formula comprising Astragali Radix, Saposhnikoviae Radix, and Atractylodis Macrocephalae Rhizoma, and is used clinically to treat renal diseases in China. Chemical metabolites in YPFP play a significant role in specific pathophysiologic mechanisms of renal diseases. Chemical metabolites, clinical application, and pharmacology of YPFP were comprehensively summarized. YPFP and its bioactive metabolites (notably flavonoids, saponins, polysaccharides, organic acids, lactones, coumarins) demonstrate significant pharmacological activities in renal diseases, including anti-fibrosis, antioxidant, immunomodulatory, anti-apoptosis, anti-inflammatory, and anti-ferroptosis. These activities alleviate symptoms and delay disease progression. Over recent decades, extensive research has confirmed the therapeutic benefits of YPFP and its bioactive metabolites in the medical intervention of renal diseases. This review systematically investigates the renoprotective effects of YPFP, including its therapeutic effect and multi-target mechanisms, providing a reference for the therapy of renal diseases.

## Graphical Abstract

**Figure:**
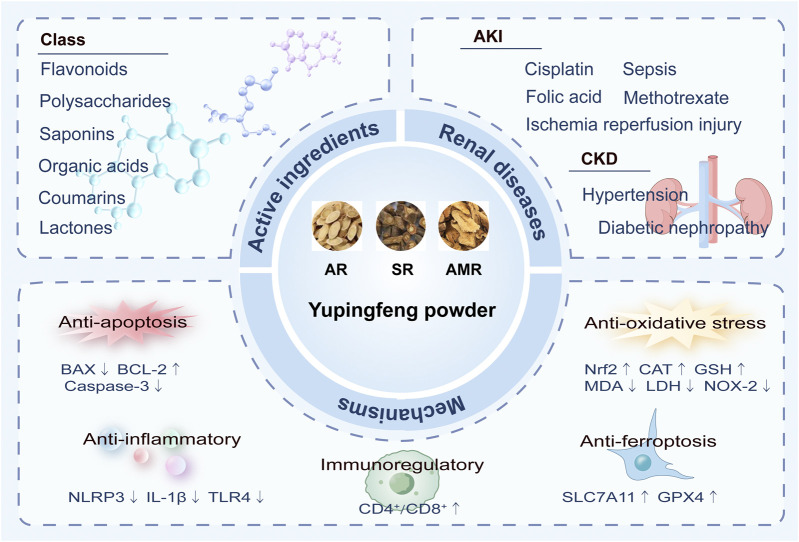

## 1 Introduction

Impaired renal function is divided into three stages: acute kidney injury (AKI), acute kidney disease (AKD), and chronic kidney disease (CKD) (Yeh et al., 2024; Zhang T. et al., 2024). AKI is an increasingly prevalent condition among hospitalized patients, characterized by a sudden decline in kidney function and associated with high incidence (Yan, 2021). Currently, there is no specific therapeutic approach available to alleviate AKI or accelerate recovery. AKI often progresses to CKD due to maladaptive repair processes. The main characteristics of CKD are a decrease in glomerular filtration rate and proteinuria. Common causes of CKD include diabetic nephropathy (DN) and hypertensive nephropathy (Girndt, 2017; Yuan et al., 2022). CKD can progress to end-stage renal disease (ESRD), requiring dialysis or kidney transplantation (Liu K. et al., 2025). The molecular mechanisms by which AKI progresses to CKD mainly involve inflammation, immunity, fibrosis, apoptosis, ferroptosis, and oxidative stress (OS) (Li et al., 2024; Yeh et al., 2024). Many animal models such as ischemia, lipopolysaccharide, cisplatin (CLP), cadmium and diabetes-induced kidney diseases have been employed to elucidate the pathological mechanisms of renal diseases (Zhang T. et al., 2024).

Yupingfeng powder (YPFP) consists of Astragali Radix (AR) (*Astragalus membranaceus* (Fisch.) Bge. var. *mongholicus* (Bge.) Hsiao or *Astragalus membranaceus* (Fisch.) Bge.), Atractylodis Macrocephalae Rhizoma (AMR) (*Atractylodes macrocephala* Koidz.) and Saposhnikoviae Radix (SR) (*Saposhnikovia divaricata* (Turcz.) Schischk.) (Bai et al., 2023; Pei et al., 2023). Chinese Pharmacopoeia (2025 edition) records dosage forms of Yupingfeng (YPF) as capsules, oral liquid, granules, and teabag (Yao et al., 2020; Zhang L. et al., 2022). YPFP has demonstrated good therapeutic effects in the treatment of Henoch-Schonlein purpura nephritis, nephrotic syndrome, and acute/chronic glomerulonephritis (Zhao and Guo, 2009). Phytochemical studies indicated that YPFP contains multiple chemical metabolites such as flavonoids (Ma et al., 2004; Huang et al., 2024), saponins (Tang et al., 2010; Li et al., 2025a), coumarins (Kim et al., 2011; Zhu H. et al., 2023), lactones (Chen et al., 2013; Sun et al., 2017; Li et al., 2025b), saccharides (Yin et al., 2019; Huang et al., 2025), and chromones. Metabolites of saponins, flavonoids, and coumarins in YPFP have been extensively studied for renal diseases (Ramazanoglu et al., 2020; Caglayan et al., 2021; Khalaf et al., 2022; Shen et al., 2023). Many flavonoids exhibit renoprotective effects in the treatment of DN, glomerulonephritis, and renal dysfunction (Vargas et al., 2018). Coumarins have inhibitory effects on loss of cell membrane integrity, apoptosis and nuclear fragmentation (Flores-Morales et al., 2023).

Previous reviews on YPFP have not provided a systematic description of its use in treating kidney diseases, merely noting in general terms that it has a beneficial therapeutic effect. This study reviews the renoprotective metabolites in YPFP and the mechanisms underlying its treatment of kidney diseases. It is hoped that the information presented in this review will provide a reliable basis for developing strategies to use YPFP and its active metabolites for the treatment of kidney diseases.

## 2 Methods

**FIGURE 1:**
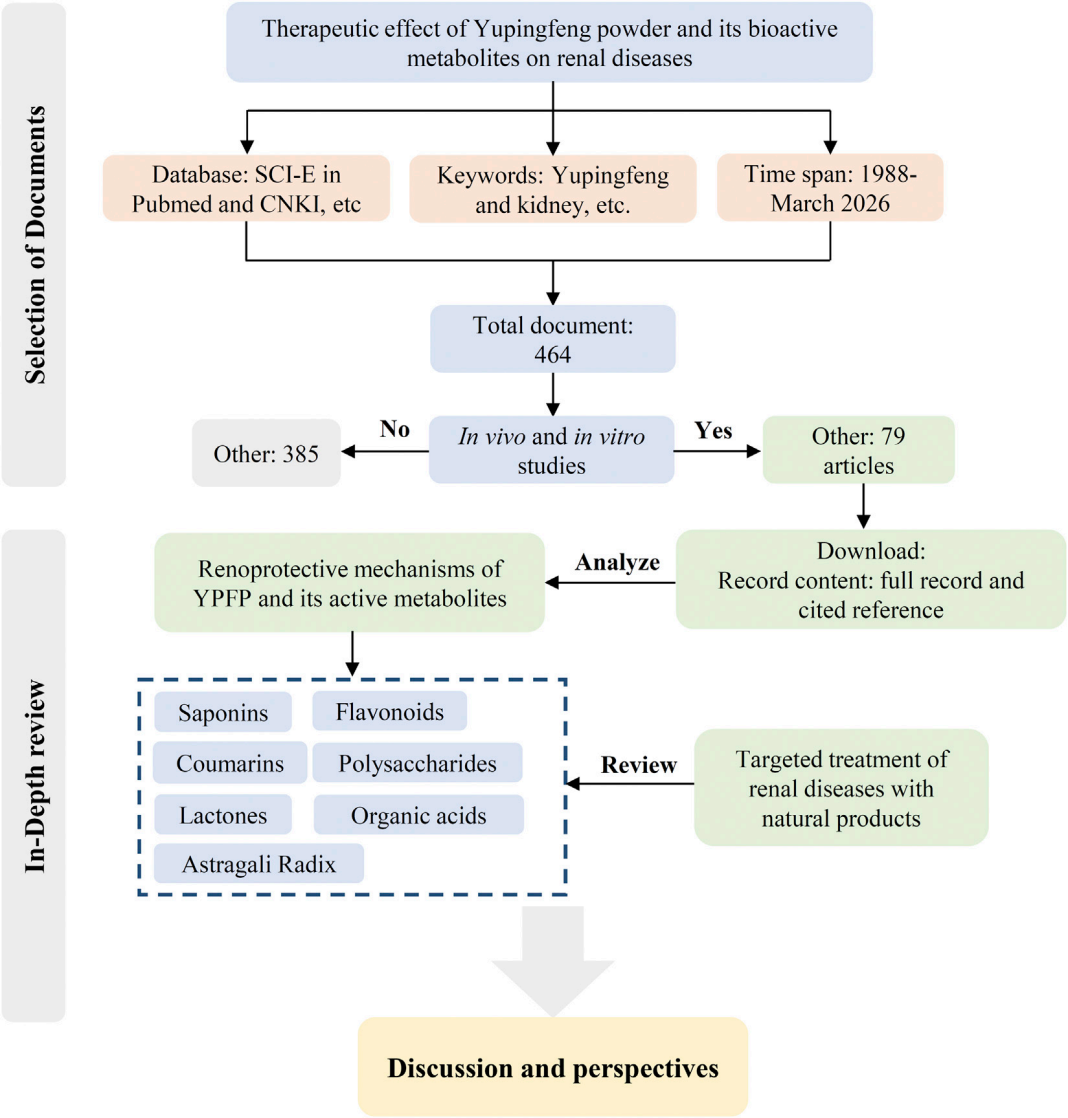
The flow chart of review.

Following PRISMA guidelines, the literature from PubMed, CNKI, Web of Science and other databases was searched and analyzed. The following keywords were used: “Astragali Radix,” “kidney,” “yupingfeng,” “Saposhnikoviae Radix,” “Atractylodis Macrocephalae Rhizoma,” “acute kidney injury,” and “chronic kidney disease.” Based on experimental and clinical studies, this study provides a comprehensive evaluation of the regulatory role of YPFP in kidney disease. Inclusion criteria were as follows: ① Studies included relevant *in vivo* and *in vitro* studies, as well as systematic reviews. ② Studies were published in Chinese or English from 1988 to March 2026. ③ Studies included systematic reviews evaluating YPFP interventions for kidney disease, covering classical formulas, single botanical drugs, extracts, and active metabolites. ④ Experimental studies identified disease or injury models, drug administration methods, routes of administration, and treatment regimens. The exclusion criteria were as follows: ① Narrative reviews. ② Network meta-analyses. ③ Repeated published literature. After analyzing the abstracts, the full text of each paper was checked. Finally, a total of 79 articles were collected for review from 464 articles identified in the initial search (Figure 1).

## 3 Historical and modern development

In 1213 A.D., YPFP was first mentioned in *Jiu Yuan Fang*, and it was used to prevent colds (Bai et al., 2023). According to TCM theory, it tonifies qi and fortifies the exterior. “Qi,” as the microscopic energy and driving force that sustains vital functions, promotes blood circulation. “Tonifying qi” promotes cellular metabolism, thereby maintaining internal balance. Literally, “fortifying the exterior” means forming a solid screen to protect the body, primarily by repairing the skin and intestinal barriers and boosting the immune system (Sun et al., 2018; He Q. et al., 2025). In both historical and modern clinical practice, YPFP formula is typically prepared as a decoction and administered orally. The historical records of YPFP in ancient texts are summarized in Table 1.

**TABLE 1:** YPFP prescriptions throughout the Chinese dynasties.

Title	Writer	Dynasty	Ratio (AR/AMR/SR)	Characteristic and/or indication
Jiu Yuan Fang	Song Zhang	Song Dynasty; 1213 A.D.	2:2:1	It is used to prevent colds
Dan Xi Xin Fa	Zhenheng Zhu	Yuan dynasty; 1347 A.D.	1:2:1	It is used to prevent colds, and treat profuse sweating
Shi Yi De Xiao Fang	Yilin Wei	Yuan dynasty; 1337 A.D.	1:2:1	It is used to treat profuse sweating
Yi Zong Jin Jian	Qian Wu	Qing dynasty; 1742 A.D.	N/A	It is used to treat profuse sweating

N/A: not applicable.

## 4 The pharmacodynamics of YPFP and its traditional medicinal plants in renal diseases

Kidney diseases mainly include AKI and CKD (Chen et al., 2024; Chen et al., 2025; Wang et al., 2025). A fairly common cause of AKI is drug therapy (Perazella and Rosner, 2022), such as calcineurin inhibitors, antibiotics, chemotherapeutic drugs, antineoplastic drugs, and contrast agents. Treatment with the antitumor drug adriamycin (ADR) causes necrosis of renal tubules and glomeruli, leading to inflammation, tubular dilation, and capillary permeability alterations. The anti-cancer chemotherapy drug cisplatin can cause severe side effects, such as nephrotoxicity characterized by tubular dysfunction. In acid urine, methotrexate and its metabolites precipitate to form free methotrexate crystals and crystal-containing casts, thereby causing AKI. Furthermore, AKI more commonly occurs due to major surgery and sepsis (Lan et al., 2023). For instance, renal ischemia reperfusion injury (IRI) is a prevalent postoperative complication that leads to CKD, ESRD, and high mortality (Geng et al., 2020). The most prevalent factor contributing to AKI in critically ill patients is sepsis, which is associated with particularly high incidence and mortality (Privratsky et al., 2023). Cecal ligation and puncture (CLAP) model in mice can induce polymicrobial sepsis and is frequently used in studies. CKD mainly includes DN, hypertensive renal damage and chronic glomerulonephritis (Hayama, 2003; Ma et al., 2024). The characteristics of DN include decreased glomerular filtration rate, diabetic glomerular lesions, and excessive urinary albumin excretion (Wal et al., 2023). Hypertensive nephropathy can induce epithelial-mesenchymal transition (EMT) and tubulointerstitial fibrosis (Costantino et al., 2021). Moreover, nephrotic syndrome (NS) is one of the common kidney diseases, and is characterized by massive proteinuria, hypoalbuminemia, generalized edema and hyperlipidemia (Agrawal et al., 2018).

In the pathogenesis of kidney diseases, stress responses such as immune response, autophagy, apoptosis, cell-cycle arrest, senescence, and inflammation play a pivotal role (Tang et al., 2023; Guo et al., 2025). When these mechanisms are disrupted, it may lead to kidney damage and trigger the development of kidney diseases (Shi et al., 2024). In recent years, the therapeutic efficacies of YPFP and its traditional medicinal plants in various AKI and CKD conditions have been confirmed by numerous studies, including nephrotic syndrome, chronic glomerulonephritis, ADR-induced renal injury, renal IRI, methotrexate-induced kidney injury, and DN.

### 4.1 Clinical application of YPFP and its traditional medicinal plants

YPFP has good therapeutic effects on chronic glomerulonephritis. YPFP was used by modern TCM practitioners to treat chronic glomerulonephritis (Zou et al., 2015). Observation of the therapeutic effect of modified YPFP on chronic glomerulonephritis revealed that modified YPFP can not only improve the clinical symptoms, but also reduce the quantity of urine protein in patients (Meng et al., 2015). Over the years, for pediatric primary nephrotic syndrome (PNS), YPF formula has been used as an ancillary therapeutic approach, resulting in elevated CD4^+^ T cells, immunoglobulin A (IgA), and immunoglobulin G (IgG) levels (Shi et al., 2018). Moreover, observational studies have shown that YPFP regulates immune function in chronic nephritis patients by increasing CD4^+^ T cell counts and the CD4^+^/CD8^+^ ratio, as well as neutrophilic granulocyte counts, while decreasing CD8^+^ T cell counts (Tao and Gao, 2011). YPFP was used to treat 27 cases of chronic renal failure complicated with infection, and the effective rate was 74%, which was similar to that of antibiotic treatment in 10 cases (Shen et al., 1988). Meanwhile, the recurrence rate of infection was low, and the improvement of the whole-body condition was obvious in YPFP group.

Medicinal plants with renoprotective action in YPFP are mainly AR, followed by AMR. Xia et al. found that in the prescriptions of 166 patients, AR and AMR were one of the core botanical drugs used to treat CKD (Xia et al., 2020). Zhang et al. suggested that adjunctive use of AR injection may effectively reduce proteinuria and creatinine in patients with DN (Zhang et al., 2019). The above results indicated that YPFP and its traditional medicinal plants have a significant improvement effect on renal diseases.

Some clinical trial data demonstrated that YPFP may be a safe and effective therapeutic TCM formula for alleviating allergic rhinitis and respiratory infection. However, in clinical research on kidney diseases, only a limited number of placebo-controlled, multi-center clinical trials, double-blind, and related randomized trials have been reported. Although numerous researchers have performed a wealth of basic animal and cell experiments on YPFP and its bioactive metabolites in the realm of renal diseases, further high-quality data from clinical trials remain necessary.

### 4.2 Preclinical studies of YPFP and its traditional medicinal plants

YPFP has been reported to treat renal diseases by improving the immune system and inhibiting inflammatory cytokines (Li et al., 2021; Li Y. et al., 2022). YPFP has a beneficial influence on CLP-induced oxidative damage to the kidneys, liver, and lungs (Zhang et al., 2012). Guo et al. showed that oral modified YPF decoction could decrease malondialdehyde (MDA) and xanthine oxidase (XOD) levels in mice with hydrocortisone-mediated renal injury (Guo et al., 2002). It was indicated that modified YPF decoction exhibited a significant antioxidant effect.

Traditional medicinal plants in YPFP and their multiple active metabolites had therapeutic effects on multiple renal diseases (including AKI, CKD, and kidney cancer), including delaying disease progression, alleviating symptoms, and improving prognosis (Hu et al., 2021; He Z. et al., 2025). Among the three traditional medicinal plants in the YPFP formula, AMR nourishes spleen and tonifies Qi, helping AR to exert its therapeutic effects (Du et al., 2013). SR expels wind-damp and relieves spasm. AR has been applied to treat renal diseases (Shi et al., 2024). Studies suggested that the AR, its extracts, and preparations play a renoprotective role. For instance, AR injections could alleviate nephrotoxicity caused by CLP and restore the metabolic disorders (Li et al., 2017). AR also plays a vital role by regulating multiple mechanisms in NS (Li A. P. et al., 2020). Moreover, Li et al. indicated that AR showed better effects in treating ADR-induced nephropathy (Li et al., 2023).

## 5 Renoprotective mechanisms of active metabolites and their traditional medicinal plants in YPFP

**FIGURE 2:**
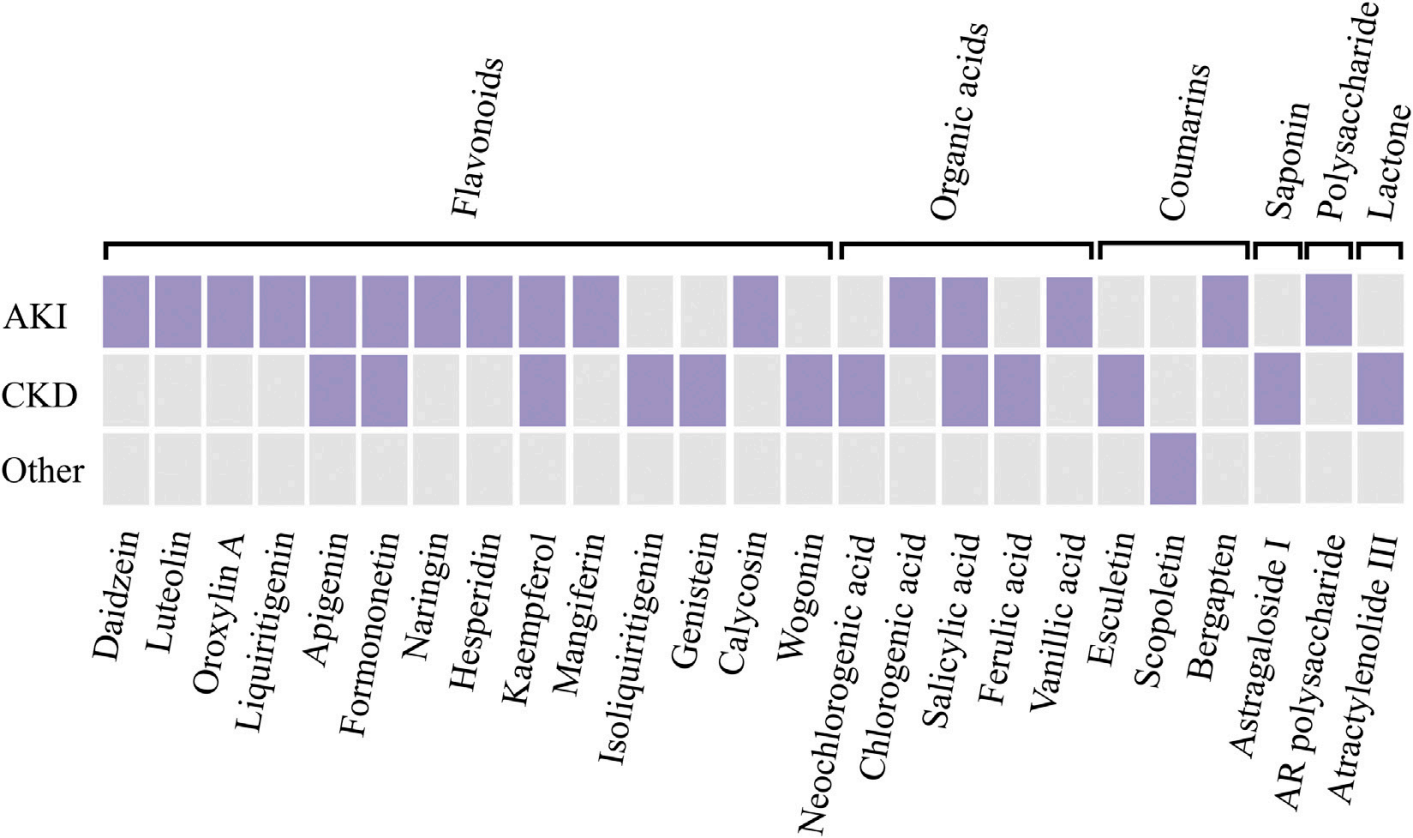
The active metabolites with kidney protective properties, and their classification.

**FIGURE 3:**
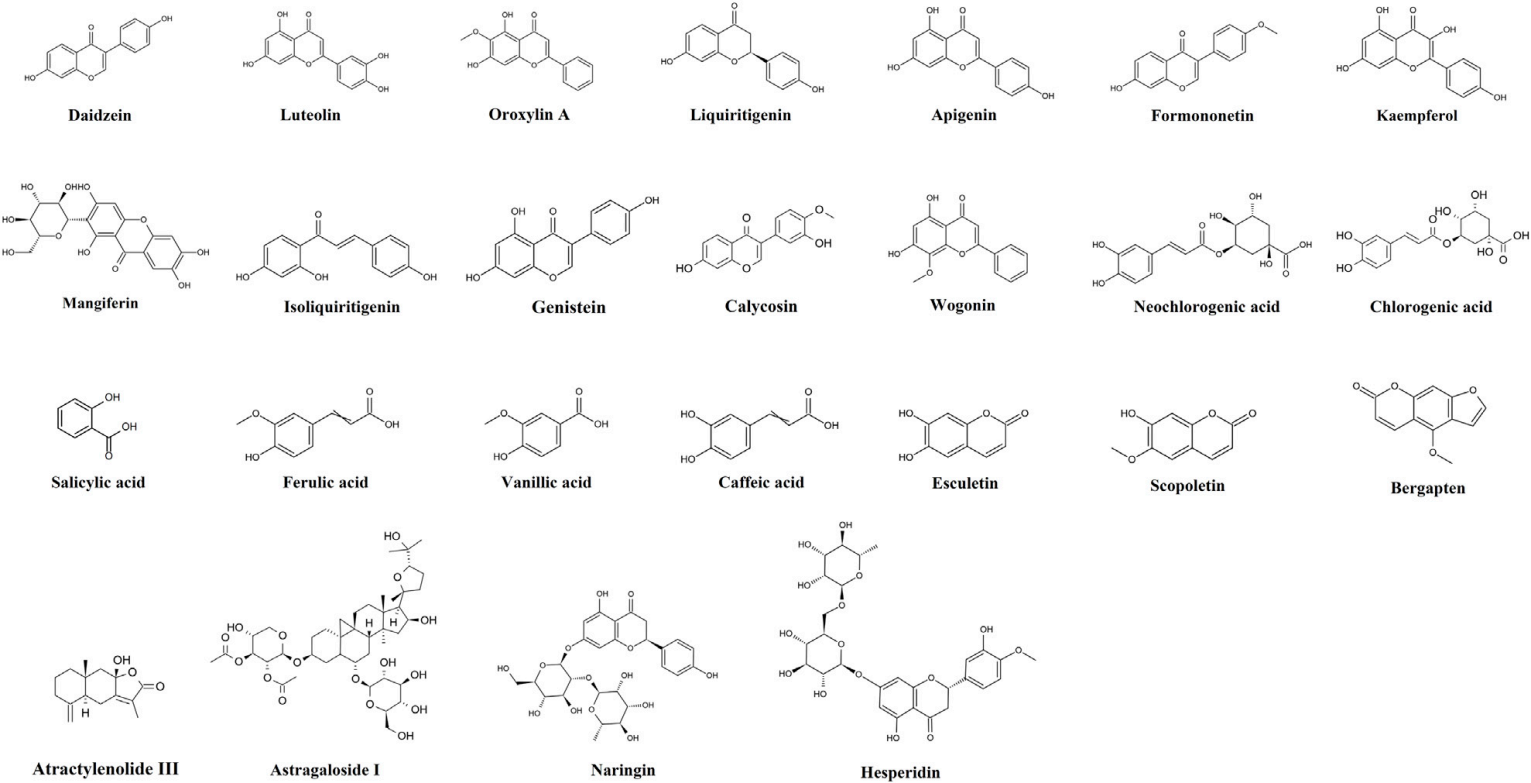
The chemical structures of active metabolites in the treatment of kidney diseases.

**FIGURE 4:**
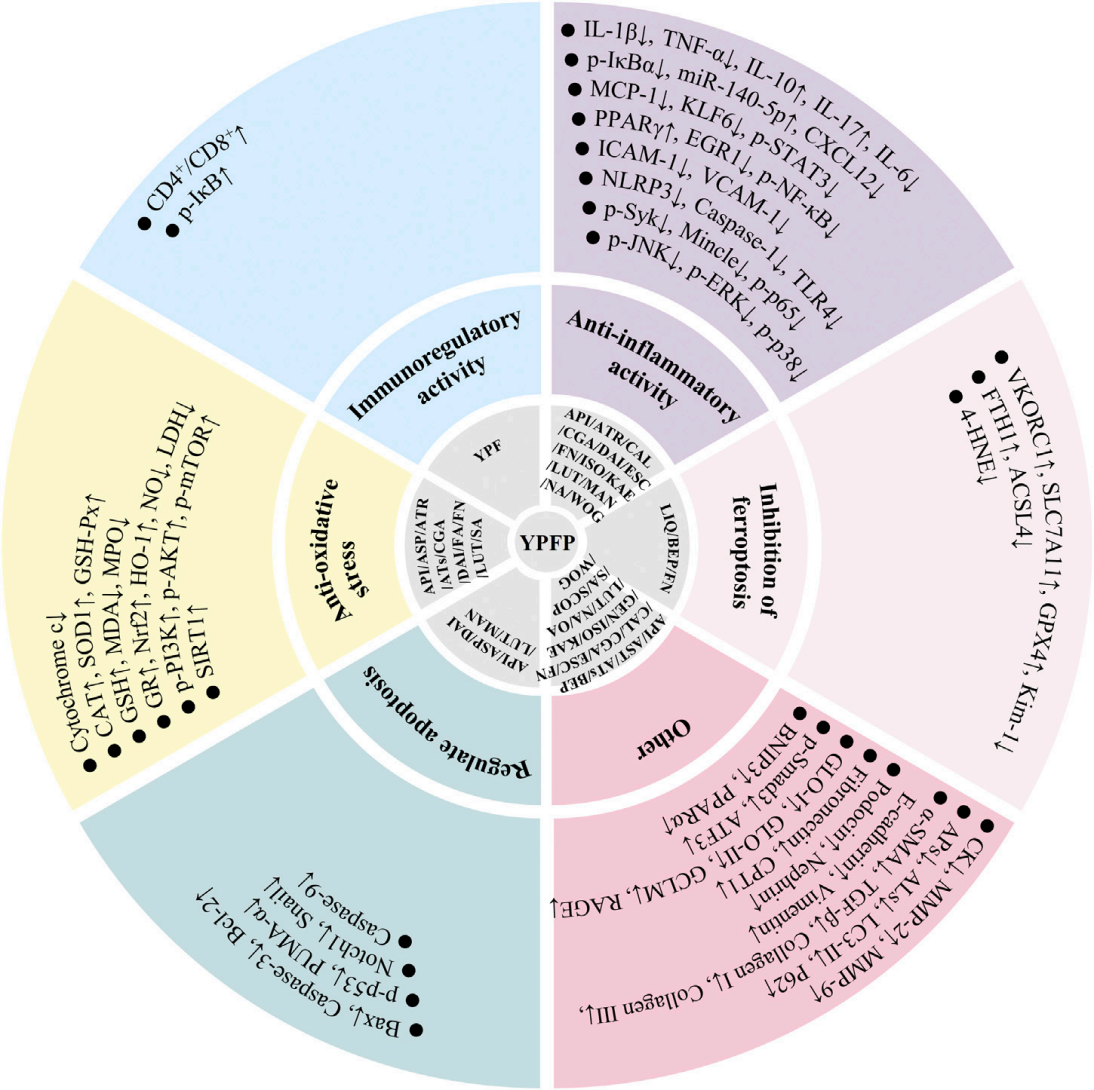
The mechanisms involved in the renoprotective action of YPFP and its active metabolites. Abbreviations: ACSL4, acyl-CoA synthetase long-chain family member 4; Als, autolysosomes; APs, autophagosomes; ATF3, activating transcription factor 3; Bax, Bcl-2-associated x protein; Bcl-2, B-cell lymphoma-2; BNIP3, Bcl-2/adenovirus E1B 19-kDa interacting protein; CAT, catalase; CK, creatine kinase; CPT1, carnitine palmitoyltransferase 1; CXCL12, C-X-C motif ligand 12; EGR1, early growth response 1; FTH1, ferritin heavy chain 1; GCLM, glutamate cysteine ligase modifier subunit; GLO-I, glyoxalase I; GLO-II, glyoxalase II; GPX4, glutathione peroxidase 4; GR, glutathione reductase; GSH, glutathione; GSH-Px, glutathione peroxidase; HO-1, heme oxygenase-1; ICAM-1, intercellular adhesion molecule 1; IL-1β, interleukin-1beta; IL-6, interleukin-6; IL-10, interleukin-10; IL-17, interleukin-17; Kim-1, kidney injury molecule 1; KLF6, Kruppel-like factor 6; LC3-II, microtubule associated protein 1 light chain 3-II; LDH, lactate dehydrogenase; MCP-1, monocyte chemoattractant protein-1; MDA, malondialdehyde; Mincle, macrophage-inducible C-type lectin; MMP-2, matrix metalloproteinase-2; MMP-9, matrix metalloproteinase-9; MPO, myeloperoxidase; NLRP3, NLR pyrin domain containing 3; NO, nitric oxide; Nrf2, nuclear factor erythroid-2-related factor 2; p-AKT, phosphorylated protein kinase B; p-ERK, phospho-extracellular signal-regulated kinase; p-IκB, phospho-inhibitor of nuclear factor-kappa B; p-IκBα, phosphorylated inhibitor of kappa B alpha; p-JNK, phospho-c-Jun N-terminal kinases; p-NF-κB, phospho-nuclear factor kappa B; PPARα, poxisome proliferator-activated receptor-alpha; PPARγ, peroxisome proliferator-activated receptor gamma; p-PI3K, phosphorylated phosphatidylinositol 3-kinase; p-STAT3, phosphorylated signal transducer and activator of transcription 3; p-Syk, phosphorylated spleen tyrosine kinase; PUMA-α, p53 upregulated modulator of apoptosis α; RAGE, receptor for advanced glycation end products; SIRT1, sirtuin 1; SLC7A11, solute carrier family 7 member 11; SOD 1, superoxide dismutase1; TGF-β, transforming growth factor beta; TLR4, toll-like receptor 4; TNF-α, tumor necrosis factor alpha; VCAM-1, vascular cell adhesion molecule-1; VKORC1, Vitamin K epoxide reductase complex subunit 1; α-SMA, alpha-smooth muscle actin; 4-HNE, 4-hydroxynonenal. Metabolites abbreviations: API, apigenin; ASP, astragalus polysaccharide; AST, astragaloside I; ATR, atractylenolide III; ATs, astragalosides; BEP, bergapten; CAL, calycosin; CGA, chlorogenic acid; DAI, daidzein; ESC, esculetin; FA, ferulic acid; FN, formononetin; GEN, genistein; ISO, isoliquiritigenin; KAE, kaempferol; LIQ, liquiritigenin; LUT, luteolin; MAN, mangiferin; NA, neochlorogenic acid; OA, oroxylin A; SA, salicylic acid; SCOP, scopoletin; WOG, wogonin.

Many of the metabolites in YPFP have renoprotective effects. Studies demonstrated that the main metabolites of YPFP with renoprotective effects include flavonoids, saponins, organic acids, polysaccharides, coumarins, and lactones (Figure 2). Various specific renoprotective metabolites include daidzein, liquiritigenin, apigenin, formononetin, calycosin, kaempferol, mangiferin, hesperidin, oroxylin A, wogonin, luteolin and naringin (flavonoids), vanillic acid, ferulic acid, neochlorogenic acid, and chlorogenic acid (organic acids), astragalosides and astragaloside I (saponins), atractylenolide III (lactone), scopoletin, and esculetin (coumarins), among others. Thus, we listed in detail three medicinal plants in YPFP and their renoprotective metabolites (Table 2). Chemical structures of these potential drugs for treating kidney diseases are displayed in Figure 3. Mechanisms for treating kidney diseases mainly include anti-inflammation, anti-renal fibrosis, anti-apoptosis, anti-ferroptosis, and anti-OS (Qu and Jiao, 2023). Renoprotective effects of YPFP and its active metabolites are based on the above mechanisms (Figure 4).

**TABLE 2:** Active ingredients for kidney treatment of YPFP.

Herbs or dosage forms	Active ingredients	Class	Model	Dosage	Result	Refs	Evidence level	Study type
Jiaweiyupingfeng decoction	N/A	N/A	*In vivo*: Hydrocortisone-mediated renal injury mice	25 g/kg/d, 10 days	MDA↓, XOD↓	Guo et al. (2002)	Medium	Experimental
AR injection	N/A	N/A	*In vivo*: Cisplatin-mediated renal injury SD rats	5 mL/kg/d, 16 days	GFR↑, Crea↓, mALB↓, Urea↓	Li et al. (2017)	Medium	Experimental
AR preparation	N/A	N/A	*In vivo*: Diabetic nephropathy patients	-	Albuminuria↓, Proteinuria↓, CRE↓	Zhang et al. (2019)	High	Experimental
AR	N/A	N/A	*In vivo*: Adriamycin-induced renal injury Sprague-Dawley rats	1.5 g/kg/d, 35 days	CPT1↓, ROS↓	Li J. et al. (2020)	Medium	Experimental
AR	N/A	Saponin	*In vivo*: Adriamycin-induced renal injury Sprague-Dawley rats	4.2 g/kg/d, 35 days	BUN↓, Scr↓, ALB↑, TG↓, TP↑, TCHO↓	Li et al. (2023)	Medium	Experimental
AR	Polysaccharide	Polysaccharide	*In vivo*: Cisplatin-mediated renal injury C57BL/6 mice *In vitro*: HK-2 cells	*In vivo*: 30 mg/kg/d, 10 days; *in vitro*: 100 μg/mL, 24 h	CRE↓, Kim-1↓, T-SOD↑, GSH-Px↑, Cytochrome-c↓	Ma et al. (2020)	Medium	Experimental
AR	Daidzein	Flavonoid	*In vivo*: Glycerol-induced acute kidney injury rats	25, 50, 100 mg/kg/d, 14 days	CRE↓, Kim-1↓, LDH↓, SOD↑, CAT↑, GSH-Px↑, GR↑, GSH↑, NO↓, MDA↓, Nfe212↑, Hmox-1↑, TNF-α↓, IL-1β↓, IL-10↑, MPO↓, NF-κB↓, Bax↓, Caspase-3↓, Bcl-2↑	Kassab et al. (2023)	Medium	Experimental
AR	Daidzein	Flavonoid	*In vivo*: Cisplatin-mediated renal injury rats	25, 50, 100 mg/kg/d, 10 days	BUN↓, CRE↓, MDA↓, SOD↑, GSH↑, TNF-α↓, IL-6↓, Bax↓, Bcl-2↑, Caspase-3↓, p-JNK↓, p-P38↓, p-ERK↓, p-NF-κB↓	Tomar et al. (2020)	Medium	Experimental
AR	Liquiritigenin	Flavonoid	*In vivo*: Folic acid-induced acute kidney injury C57BL/6 mice *In vitro*: HK-2 cells	*In vivo*: 15 mg/kg/d, 3 days; *In vitro*: 10 μM	CRE↓, BUN↓, E-cadherin↑, Kim-1↓, TFR1↓, SLC7A11↑, GPX4↑, MDA↓, GSH↑, VKORC1↑	Guo et al. (2024)	Medium	Experimental
AR	Apigenin	Flavonoid	*In vivo*: Renal ischemia reperfusion injury C57BL/6 mice	5, 10, 20 mg/kg	Caspase-9↓, Caspase-3↓, Bax↓, Bcl-2↑, TNF-α↓, IL-1β↓, IL-6↓, p-IκBα↓, p65↓, CXCL12↓, miR-140-5p↑, BUN↓, Scr↓	He et al. (2021)	Medium	Experimental
AR	Apigenin	Flavonoid	*In vivo*: Methotrexate-induced kidney toxicity CD-1 mice	3 mg/kg/d, 4 days	CRE↓, SOD1↑, CAT↑, GSH-Px↑, GSH↑, MDA↓	Sahindokuyucu-Kocasari et al. (2021)	Medium	Experimental
AR	Apigenin	Flavonoid	*In vivo*: Deoxycorticosterone acetate-salt induced hypertension male Sprague-Dawley rat	0.2% apigenin diet	TGF-β1↓, p-SMAD2/3↓, CTGF↓, COL1a1↓, COL3a1↓, p-MAPK↑, SIRT1↑, TRPV4↓	Wei et al. (2017)	Medium	Experimental
AR	Formononetin	Flavonoid	*In vivo*: Renal ischemia reperfusion injury C57BL/6 mice	12.5, 25, 50 mg/kg/d, 7 days	Scr↓, BUN↓, Kim-1↓, NGAL↓, MCP-1↓, TNF-α↓, IL-1β↓, iNOS↓, p-STAT3↓, KLF6↓	Zhang X. et al. (2024)	Medium	Experimental
AR	Calycosin	Flavonoid	*In vivo*: Renal ischemia reperfusion injury C57BL/6 mice	5, 10, 20 mg/kg/d, 7 days	CRE↓, Urea↓, Kim-1↓, IL-1β↓, IL-6↓, TNF-α↓, p-IκBα↓, p-NF-κB↓, EGR1↓, PPARγ↑, HIF-1α↓	Zhang N. et al. (2022)	Medium	Experimental
AR	Calycosin	Flavonoid	*In vivo*: Adriamycin-induced renal injury Sprague-Dawley rats	10, 20 mg/kg/d, 28 days	BUN↓, Scr↓, UAE↓, TG↓, TC↓, Nephrin↑, Podocin↑, Notch1↓, Snail↓, Vimentin↓, α-SMA↓	Ma et al. (2025)	Medium	Experimental
AR	Kaempferol	Flavonoid	*In vivo*: Cecal ligation and puncture injury ICR mice	1 mg/kg	IL-1β↓, COX-2↓, TNF-α↓, ICAM-1↓, VCAM-1↓, MCP-1↓	Xu et al. (2023)	Medium	Experimental
AR	Mangiferin	Flavonoid	*In vivo*: Cecal ligation and puncture injury C57BL/6 mice	15 mg/kg	Caspase-9↓, Caspase-3↓, NLRP3↓, BUN↓, Cre↓, MDA↓, IL-1β↓, IL-18↓, Nrf2↑	He et al. (2014)	Medium	Experimental
AR	Oroxylin A	Flavonoid	*In vivo*: Renal ischemia reperfusion injury C57BL/6J mice	20 mg/kg	Bcl-2↑, Scr↓, BUN↓, Kim-1↓, Bax↓, BNIP3↑, PPARα↑, STAT3↓, HIF1α↑, Fibronectin↓, α-SMA↓	Yao et al. (2022)	Medium	Experimental
AR	Naringin	Flavonoid	*In vivo*: Renal ischemia reperfusion injury Wistar rats	50, 100 mg/kg	HIF-1α↑, Caspase-3↓, IL-1β↓	Danis et al. (2024)	Medium	Experimental
AR/SR	Vanillic acid	Organic acid	*In vivo*: Methotrexate-induced kidney toxicity Wistar rats	25, 50, 100 mg/kg/d, 7 days	BUN↓, CRE↓, MDA↓, TAC↑, TNF-α↓, IL-1β↓, Nrf2↑, Bcl-2↑, Caspase-3↓, Bax↓	Amini et al. (2024)	Medium	Experimental
AR	Ferulic acid	Organic acid	*In vivo*: Nomega-nitro-L-arginine methyl ester induced hypertensive Wistar rats	0.8 g/kg	LDH↓, CRE↓, SOD↑	Alam et al. (2013)	Medium	Experimental
AR	Isoliquiritigenin	Flavonoid	*In vivo*: Unilateral ureteral obstruction C57BL/6 mice	7.5, 30 mg/kg	TNF-α↓, IL-1β↓, IL-6, MCP-1↓, BUN↓, Scr↓, iNOS↓, p-p65↓, p-Syk↓, Mincle↓, α-SMA↓	Liao et al. (2020)	Medium	Experimental
AR	Formononetin	Flavonoid	*In vivo*: High fat diet and low dose of streptozotocin induced Sprague-Dawely rats	10, 20, 40 mg/kg/d, 16 weeks	GSH↑, SOD↑, SIRT1↑	Oza and Kulkarni (2019)	Medium	Experimental
AR	Formononetin	Flavonoid	*In vivo*: Unilateral ureteral obstruction induced C57BL/6 mice	20 mg/kg/d, 28 days	BUN↓, CRE↓, Kim-1↓, Fibronectin↓, Col1a1↓, α-SMA↓, SLC7A11↑, GPX4↑, 4-HNE↓, p-Smad3↓, ATF3↓	Zhu B. et al. (2023)	Medium	Experimental
AR	Hesperidin	Flavonoid	*In vivo*: Renal ischemia reperfusion injury rats	50, 100 mg/kg	TAS↑, SOD↑, TOS↓, MDA↓, OSI↓, MPO↓	Karacaer et al. (2023)	Medium	Experimental
AR	Astragalosides	Saponin	*In vitro*: Adriamycin-induced podocyte injury	50, 100, 200 μg/mL	LDH↓, MDA↓, SOD↑, MMP-9↑, MMP-2↑	Sai et al. (2018)	Low	Experimental
AR	Astragaloside I	Saponin	*In vivo*: C57BLKS/J db/db mice	10, 20, 40 mg/kg/d, 8 weeks	UAER↓, UACR↓, BUN↓, Scr↓, Col-1↓, Col-4↓, α-SMA↓, Fibronectin↓, E-cadherin↑, TNF-β1↓, p-Smad2↓, p-Smad3↓, HDAC3↓, Klotho↑	Zhang X. et al. (2024)	Medium	Experimental
AMR	Luteolin	Flavonoid	*In vivo*: Adriamycin-induced renal injury Wistar rats	50, 100 mg/kg, 14 days	LDH↓, CRE↓, Urea↓, GSH-Px↑, GSH↑, GST↑, SOD↑, CAT↑, TSH↑, LPO↓, RONS↓, XO↓, NO↓, MPO↓, TNF-α↓, IL-1β↓, IL-10↑, Caspase-3↓, Caspase-9↓	Owumi et al. (2021)	Medium	Experimental
AMR	Luteolin	Flavonoid	*In vivo*: Cisplatin-mediated renal injury C57BL/6 mice	5, 50, 100 mg/kg/d, 3 days	BUN↓, CRE↓, GSH↑, SOD↑, Catalase↑, p-p53↓, PUMA-α↓, Bax↓, Bcl-2↑, Bcl-xL↑, Caspase-3↓	Kang et al. (2011)	Medium	Experimental
AMR	Luteolin	Flavonoid	*In vivo*: Methotrexate-induced kidney toxicity Wistar Albino rats	50 mg/kg/d, 14 days	Urea↓, CRE↓, MDA↓, GSH↑, SOD↑, CAT↑, GSH-Px↑, GR↑, NF-κB↓, TNF-α↓, IL-1β↓, Bcl-2↑, Bax↓, Nrf2↑	Dar et al. (2021)	Medium	Experimental
SR	Wogonin	Flavonoid	*In vivo*: Streptozotocin-induced C57/BL mice	10, 20, 40 mg/kg/d, 8 weeks	CRE↓, BUN↓, Kim-1↓, TNF-α↓, IL-1β↓, MCP-1↓, LC3I/II↑, P62↓, Beclin 1↑, Atg7↑, Col-I↓, E-cadherin↑, α-SMA↓, p-PI3K↓, p-Akt↓, p-mTOR↓, p- NF-κB↓	Lei et al. (2021)	Medium	Experimental
AMR	Atractylenolide III	Lactone	*In vivo*: 5/6 nephrectomy Sprague-Dawley rats	2.4 mg/kg/d, 5 weeks	BUN↓, Scr↓, TNF-α↓, IL-1β↓, IL-6↓, SOD↑, GSH-Px↑, CAT↑, MDA↓	Wang et al. (2019)	Medium	Experimental
AMR	Chlorogenic acid	Organic acid	*In vivo*: C57BL/6 mice kidney fibrosis model by unilateral uretera obstruction	40, 80 mg/kg/d, 10 days	Collagen I↓, α-SMA↓, MCP-1↓, NLRP3↓, iNOS↓, Caspase-1↓, ROS↓, MDA↓, GSH-Px↑, SOD↑, Fibronectin↓, Collagen I↓, TGF-β1↓, TLR4↓, p- NF-κB↓	Jiao et al. (2024)	Medium	Experimental
AMR	Neochlorogenic acid	Organic acid	*In vivo*: Db/db mice	100 mg/kg/d, 12 weeks	BUN↓, CRE↓, p- NF-κB↓, p-IκB↓, TNF-α↓, MCP-1↓	Hung et al. (2023)	Medium	Experimental
SR	Scopoletin	Coumarin	*In vitro*: D-glucose induced HK-2 cells	5,10,20 μg/mL, 2 h	TGF-β↓, α-SMA↓, Collagen I↓, Collagen III↓, E-cadherin↑, Vimentin↓	Kundu et al. (2024)	Low	Experimental
SR	Kaempferol	Coumarin	*In vivo*: Spontaneously hypertensive rats *In vitro*: HK-2 cells	*In vivo*: 10, 20, 40 mg/kg/d, 12 weeks, *In vitro*: 5,10,20 μM	CRE↓, BUN↓, TNF-α↓, IL-1β↓, MCP-1↓, Collagen I↓, Collagen III↓, TGF-β1↓, α-SMA↓, PCNA↓, Shh↓, Ptch1↑, Smo↓, Sufu↑, Gli1↓, Gli2↓, E-cadherin↑, N-cadherin↓	Guan et al. (2023)	Medium	Experimental
SR	Esculetin	Coumarin	*In vivo*: Streptozotocin-induced Wistar rats	50 mg/kg/d, 4 days	BUN↓, Kim-1↓, Parkin↑, PINK1↑, LC3I/II↑, p- NF-κB↓, p62↓, TLR4↓, TNF-α↓, MCP-1↓, Caspase-3↓, Caspase-7↓	Dagar et al. (2025)	Medium	Experimental

N/A: not applicable.

### 5.1 Immunoregulatory activity

The recruitment of innate immune cells to the site of injury represents the initial manifestation of the early inflammatory response in AKI. The surface markers of CD4^+^ and CD8^+^ serve as indicators of T cells maturation. The imbalance in the CD4^+^/CD8^+^ T cell ratio is primarily associated with immune system dysfunction (Losappio et al., 2020). It has been reported that T cells, particularly CD4^+^ cells, function as mediators in AKI (Martina et al., 2014). Moreover, advanced immunological alterations during the progression of CKD included elevated CD4^+^ infiltration and enhanced IL-1β levels. The CD4^+^ T cell population in the kidney is composed of Th cells and Treg cells, among which Th cell clones may be further subdivided into three major subsets: Th1 cells, Th2 cells, and Th17 cells. Th17 cells secrete interleukin 17 (IL-17) (Lee et al., 2024).

TCM formula of YPFP has been used to treat immune-related diseases (Gao et al., 2009; Nikles et al., 2017; Zhou et al., 2019; Xu and Song, 2025). For instance, YPF upregulated splenic lymphocytes CD4^+^/CD8^+^ ratio induced by *Candida* albicans and increased the levels of IL-17 (Li Y. et al., 2022).

### 5.2 Anti-oxidative stress

**FIGURE 5:**
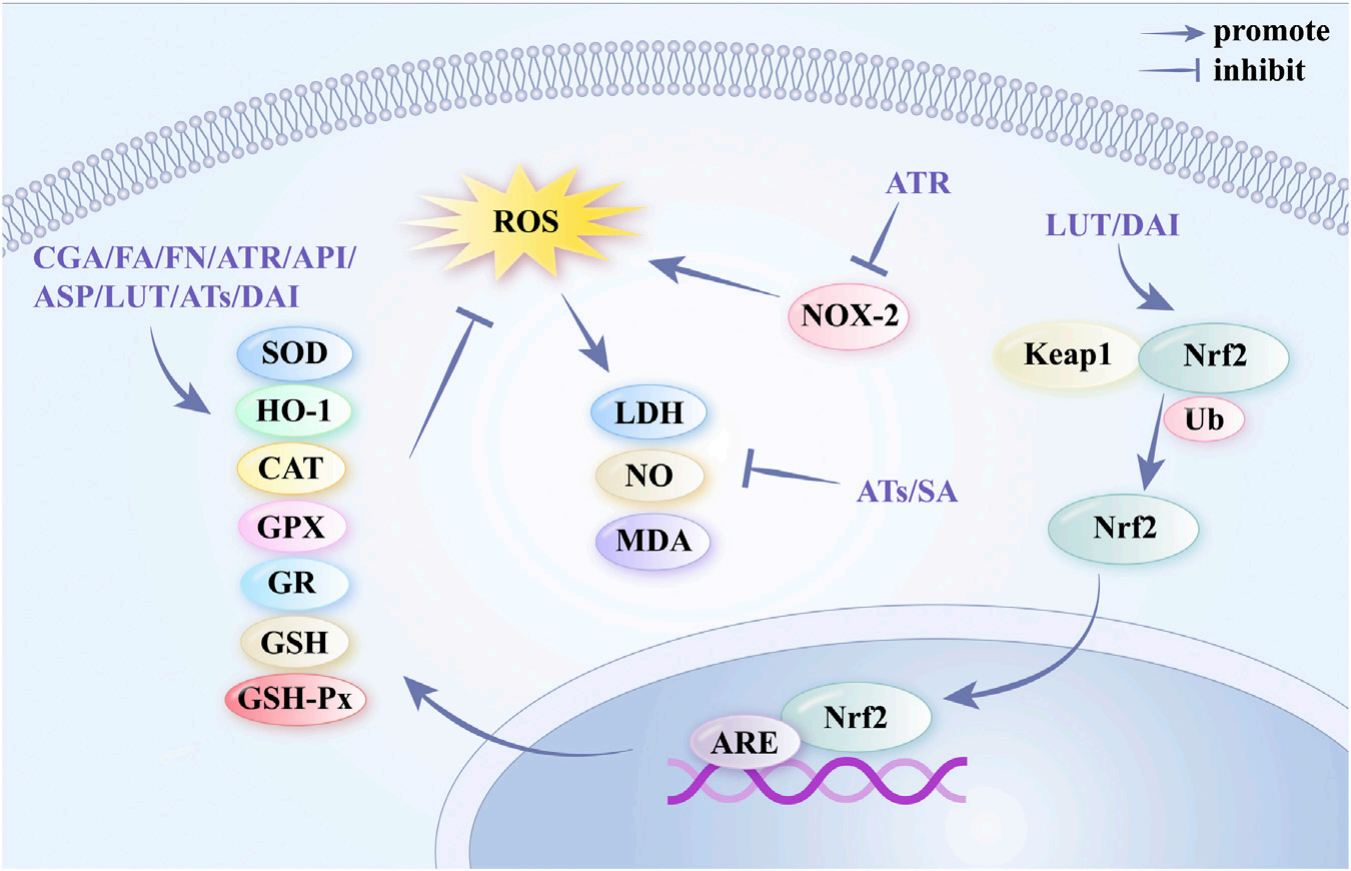
The anti-oxidative stress mechanisms of YPFP in kidney diseases. CGA, FA, FN, ATR, API, ASP, and ATs increase the activities of antioxidant enzymes SOD, HO-1, CAT, GPX, GR, GSH and GSH-Px; LUT and DAI activate Nrf2 and leading to ROS reduction. ATR inhibits NOX-2, leading to a reduction in ROS. Abbreviations: API, apigenin; ARE, antioxidant response elements; ASP, astragalus polysaccharide; ATR, atractylenolide III; ATs, astragalosides; CAT, catalase; CGA, chlorogenic acid; DAI, daidzein; FA, ferulic acid; FN, formononetin; GPX, glutathione peroxidase; GR, glutathione reductase; GSH, gutathione; GSH-Px, glutathione peroxidase; HO-1, heme oxygenase-1; LDH, lactate dehydrogenase; LUT, luteolin; MDA, malondialdehyde; NO, nitric oxide; NOX-2, NADPH oxidase 2; Nrf2, nuclear factor erythroid 2-related factor 2; ROS, reactive oxygen species; SA, salicylic acid; SOD, superoxide dismutase; Keap 1, kelch-like ECH-associated protein 1.

The overproduction of reactive oxygen species (ROS) and the impairment of antioxidant mechanisms are critical factors in the onset and progression of kidney diseases. OS triggers conformational alterations in Keap1, which rescues nuclear factor erythroid 2-related factor 2 (Nrf2) from degradation in the proteasome and enables its translocation to the nucleus (Østergaard et al., 2020). Furthermore, the activation of NADPH oxidase 2 (NOX-2) can lead to the production of ROS (Figure 5). Nrf2 plays a critical role in maintaining cellular redox homeostasis through upregulation of glutathione peroxidase (GSH-Px), superoxide dismutase (SOD), and heme oxygenase-1 (HO-1). In addition, the renoprotective effects of Nrf2 extend beyond antioxidant defense, encompassing its anti-inflammatory, anti-apoptosis, and anti-fibrosis properties. On the one hand, Nrf2 suppresses inflammation indirectly by reducing ROS and inhibiting nuclear factor kappa B (NF-κB) signaling (Ng et al., 2024). On the other hand, Nrf2 activation alleviates fibrosis by interacting with transforming growth factor-beta (TGF-β) signaling. In terms of anti-apoptosis, Nrf2 induces antioxidant and detoxifying genes such as HO-1 and GSH-Px, indirectly restores the B-cell lymphoma-2 (Bcl-2)/Bax ratio, and inhibits caspase activity (Li et al., 2026).

AR polysaccharide attenuated oxidative damage by reducing the generation of ROS and improving the activities of GSH-Px and total SOD (Ma et al., 2020). Pretreatment with apigenin alleviated renal toxicity by decreasing creatinine and MDA levels and increasing catalase (CAT), glutathione (GSH), superoxide dismutase 1 (SOD1) and GSH-Px levels (Sahindokuyucu-Kocasari et al., 2021). Astragalosides maintained OS homeostasis in MPC-5 podocytes with ADR injury by inhibiting matrix metalloproteinase (MMP)-9 and MMP-2 expression (Sai et al., 2018). Daidzein inhibited oxidative damage and protected against AKI by increasing GSH-Px, CAT, SOD, glutathione reductase (GR), and GSH levels, and reducing nitric oxide (NO) and MDA levels (Kassab et al., 2023). After treating 5/6 nephrectomy model rats with atractylenolide III, it was observed that mitochondrial dilation was weakened, the activities of CAT, GSH-Px, and SOD were enhanced, and MDA levels were reduced (Wang et al., 2019). In the diabetic animal model, formononetin alleviated OS by elevating the GSH and SOD levels (Oza and Kulkarni, 2019). Scopoletin could alleviate the production of ROS caused by high glucose, reduce lipid peroxidation, and restore the levels of GSH in human kidney-2 (HK-2) cells (Kundu et al., 2024). Salicylic acid had a renoprotective effect against oxidative damage caused by gentamicin (Zaidi and Usman, 2021). Liquiritigenin markedly alleviated the loss of renal function through inhibiting mitochondrial morphological changes and upregulated glutathione peroxidase-4 (GPX-4) and GSH levels, while downregulated MDA and divalent iron levels in a folic acid (FA)-mediated AKI model (Guo et al., 2024). Additionally, hesperidin exerted protective effects on the renal tissues of renal IRI mice by increasing SOD levels and decreasing MDA levels (Karacaer et al., 2023). Luteolin, as an antioxidant trigger, could decrease MDA levels, elevate GSH levels, enhance antioxidant enzyme activities, and elevate the *Nrf2* expression (Dar et al., 2021). The treatment with luteolin could also prevent ADR-induced renal oxidative damage by reducing NO and lactate dehydrogenase (LDH) levels, and increasing SOD, GSH-Px, GSH, glutathione-S-transferase (GST), and CAT levels (Owumi et al., 2021). In the podocyte model subjected to damage induced by ADR, astragalosides reduced the levels of LDH and MDA, and increased SOD levels (Sai et al., 2018). Ferulic acid therapy could improve the function of the kidneys by reducing LDH expression and increasing SOD levels (Alam et al., 2013).

### 5.3 Regulate apoptosis

**FIGURE 6:**
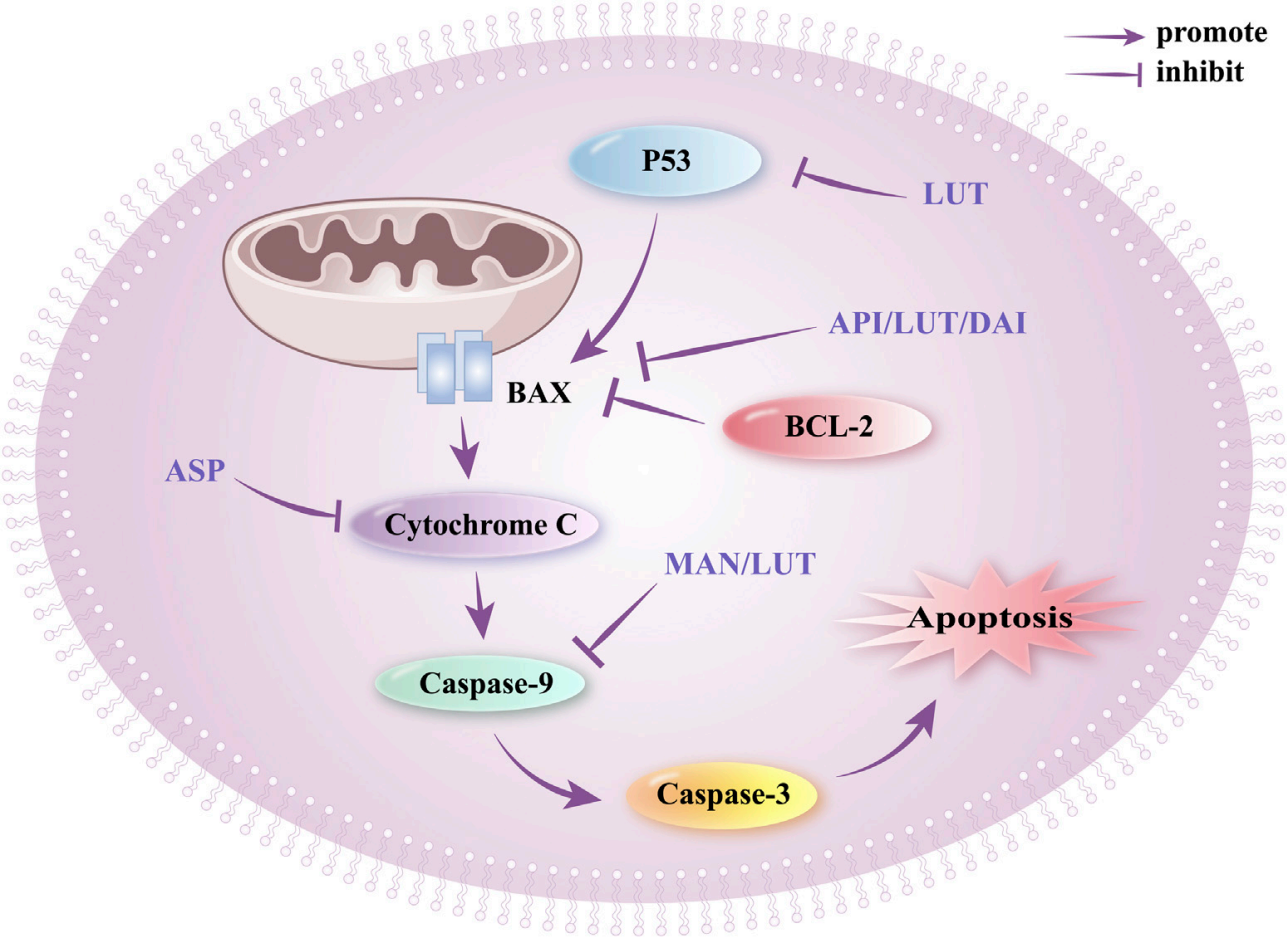
The anti-apoptosis mechanisms of YPFP in kidney diseases. LUT inhibits p53 and its phosphorylation levels, as well as suppressing BAX, and caspase-3 activity; DAI, API and LUT inhibit BAX and caspase-3 expression; MAN inhibits caspase-3 and caspase-9 expression, ultimately alleviating apoptosis. Abbreviations: API, apigenin; ASP, astragalus polysaccharide; BAX, B-cell lymphoma-2-associated x protein; BCL-2, B-cell lymphoma-2; DAI, daidzein; LUT, luteolin; MAN, mangiferin.

In the AKI models, renal tubular cells may undergo apoptotic death during the processes of development and regeneration (Maremonti et al., 2022; Sanz et al., 2023). Regarding apoptosis, mitochondria establish and maintain a permissive environment that supports pro-death signaling pathways. This results in the loss of outer mitochondrial membrane integrity, subsequently releasing intermembrane proteins, thereby activating intracellular death signals (Khatun et al., 2024). In the mitochondrial pathway, stress-induced signals promote cytochrome c release from mitochondria via Bcl-2 proteins (Nowak and Edelstein, 2020). Moreover, cytochrome c binds to the cytoplasmic protein apoptotic protease-activating factor 1 (Apaf-1), promoting the formation of the apoptosome. Once the apoptosome is formed, it recruits and activates caspase-9, which in turn activates caspase-3 (Figure 6).

Daidzein could mitigate kidney damage through downregulation of Bax and caspase-3 expression while simultaneously promoting Bcl-2 expression (Tomar et al., 2020). Moreover, luteolin improved CLP-induced nephrotoxicity by inhibiting p53 and its phosphorylation levels, as well as suppressing PUMA-α, Bax, and caspase-3 activity (Kang et al., 2011). Luteolin alleviated methotrexate (MTX)-induced toxicity by upregulating Bcl-2 and downregulating the Bax and caspase-3 protein levels (Dar et al., 2021). The treatment with luteolin could prevent ADR-induced renal apoptosis damage by reducing caspase-9 and caspase-3 levels (Owumi et al., 2021). Vanillic acid possessed anti-apoptotic properties that can prevent MTX-induced nephrotoxicity. This is manifested by downregulation of *caspase-3* and *Bax* mRNA levels, while simultaneously the upregulation of *Bcl-2* mRNA level (Amini et al., 2024). Calycosin could exert a renoprotective effect on ADR-induced rats by alleviating podocyte damage through suppression of the Notch1/Snail pathway (Ma et al., 2025). Mangiferin inhibited apoptosis in a mouse CLAP injury model via decreasing the caspase-3 and caspase-9 levels (He et al., 2014).

### 5.4 Inhibition of ferroptosis

**FIGURE 7:**
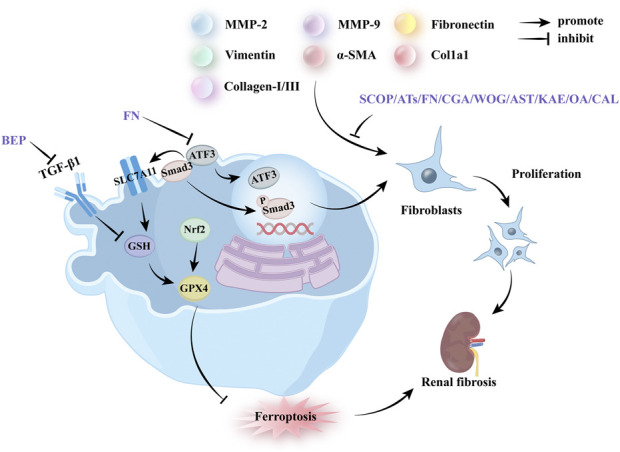
The ferroptosis and renal fibrosis mechanisms of YPFP in kidney diseases. FN inhibits ferroptosis-mediated fibrosis via suppressing the Smad3/ATF3/SLC7A11 pathway. BEP protects against fibrosis caused by TGF-β1 and ferroptosis induced by GPX4. SCOP, ATs, FN, CGA, WOG, AST, KAE, OA, and CAL alleviate renal fibrosis via decreasing the fibrosis markers of α-SMA and collagen-III, et al. Abbreviations: AST, astragaloside I; ATF3, activating transcription factor 3; ATs, astragalosides; BEP, bergapten; CAL, calycosin; CGA, chlorogenic acid; FN, formononetin; GPX4, glutathione peroxidase 4; GSH, glutathione; KAE, kaempferol; MMP-2, matrix metalloproteinase-2; MMP-9, matrix metalloproteinase-9; Nrf2, nuclear factor erythroid 2-related factor 2; OA, oroxylin A; SCOP, scopoletin; SLC7A11, solute carrier family 7 member 11; Smad 3, small mother against decapentaplegic family member 3; TGF-β1, transforming growth factor-beta1; WOG, wogonin; α-SMA, alpha-smooth muscle actin.

Ferroptosis has been identified in a variety of renal diseases, including DN, renal cell carcinoma, and AKI (Tang and Xiao, 2020; Wang et al., 2023). System Xc-is an amino acid antiporter, which is a heterodimer composed of two subunits, solute carrier family 7 member 11 (SLC7A11) and SLC3A2. Cystine and glutamate are exchanged in and out of the cell by system Xc-at a ratio of 1:1. Inhibiting the absorption of cystine affects a decrease in system Xc-activity, resulting in reduced GSH synthesis and impaired cellular antioxidant capacity, ultimately triggering oxidative damage and ferroptosis (Li J. et al., 2020) (Figure 7).

Liquiritigenin could improve renal function by increasing the vitamin K epoxide reductase complex subunit 1 (VKORC1), SLC7A11 and GPX-4 protein and decreasing the expression of kidney injury molecule-1 (Kim-1) (Guo et al., 2024). Li et al. revealed that the nephroprotective function of bergapten may be related to the suppression of ferroptosis (Li L. et al., 2025). Bergapten holds promise for development as a drug to treat renal fibrosis. Formononetin may inhibit ferroptosis-mediated fibrosis via suppressing the Smad3/ATF3/SLC7A11 pathway, thus ameliorating CKD (Zhu B. et al., 2023).

### 5.5 Anti-inflammatory activity

**FIGURE 8:**
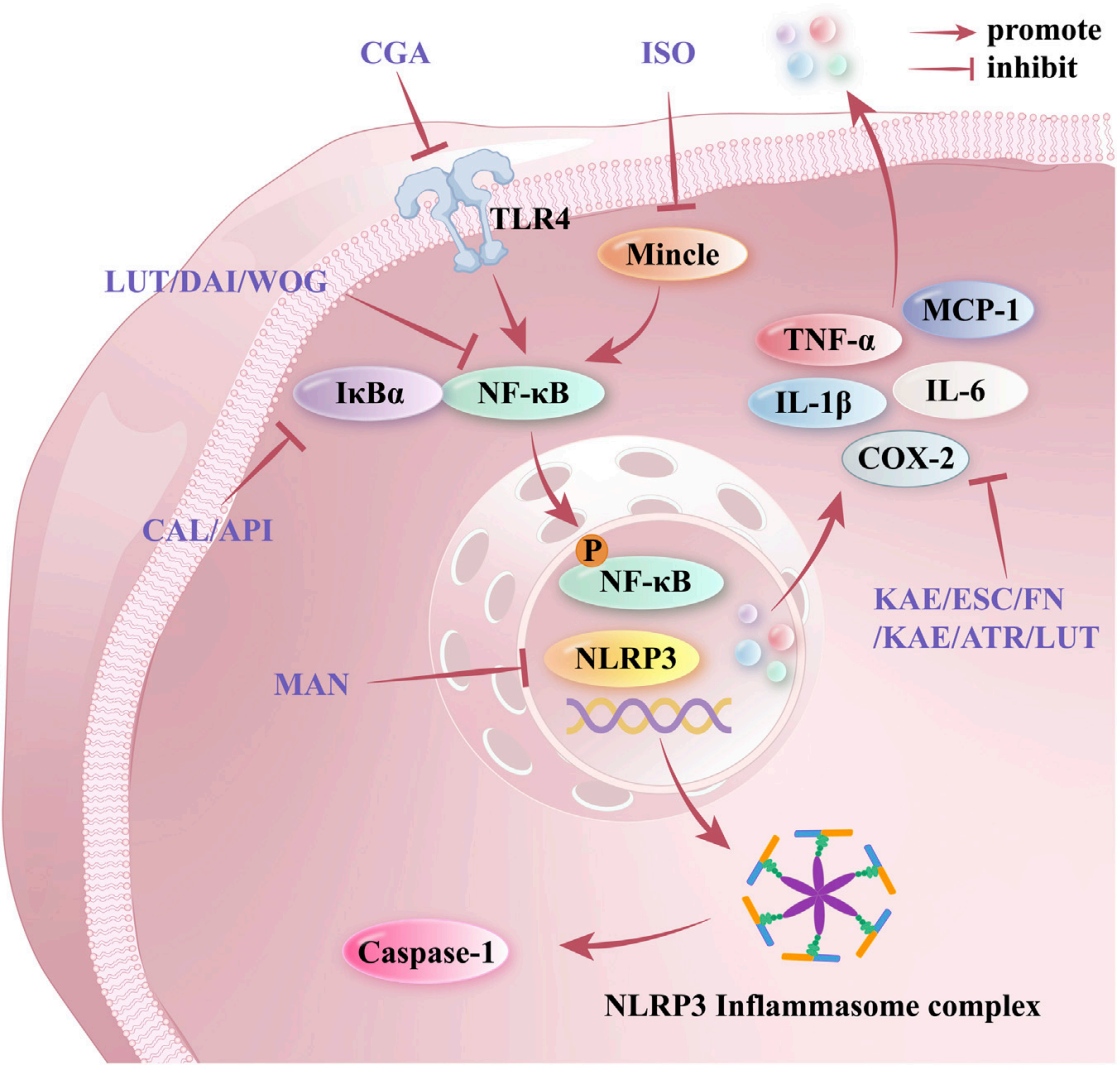
The anti-inflammation mechanisms of YPFP in kidney diseases. MAN inhibits NLRP3 inflammasome activation, thereby reducing IL-1β and IL-18 levels; CGA regulates TLR4/NF-κB signaling pathways, ISO regulates the Mincle/Syk/NF-κB pathway, KAE, ESC, FN, KAE, ATR and LUT inhibit the levels of TNF-α, IL-6, and IL-1β, et al. API and CAL suppressed IκBα phosphorylation, thereby preventing NF-κB from entering the cell nucleus. These pathways finally alleviated renal inflammation. Abbreviations: API, apigenin; ATR, atractylenolide III; CAL, calycosin; CGA, chlorogenic acid; COX-2, cyclooxygenase-2; DAI, daidzein; ESC, esculetin; FN, formononetin; IL-1β, interleukin-1beta; IL-6, interleukin-6; ISO, isoliquiritigenin; IκBα, inhibitor of NF-kappa B alpha; KAE, kaempferol; LUT, luteolin; MAN, mangiferin; MCP-1, monocyte chemoattractant protein-1; Mincle, macrophage-inducible C-type lectin; NA, neochlorogenic acid; NF-κB, nuclear factor kappa B; NLRP3, NLR pyrin domain containing 3; TLR4, toll-like receptor 4; TNF-α, tumor necrosis factor alpha; WOG, wogonin.

Tumor necrosis factor alpha (TNF-α) induces the production of ROS, which subsequently activates the transcription factor NF-κB. NF-κB then stimulates the release of pro-inflammatory cytokines including TNF-α, ultimately exacerbating kidney damage (Fang et al., 2021). Moreover, intercellular adhesion molecule 1 (ICAM-1) and VCAM-1 facilitate the inflammatory response and drive leukocyte migration during the inflammatory process. These two factors play significant roles in regulating the pathological state of kidney diseases (Singh et al., 2023). Activation of the NOD-like receptor family pyrin domain containing 3 (NLRP3) inflammasome by multiple factors triggers an inflammatory cascade, ultimately causing the activation of caspase-1 and the generation of interleukin-1beta (IL-1β) and IL-18. (Jiang et al., 2024) (Figure 8).

CLAP injury and IRI significantly affect renal metabolism, triggering renal injury and pro-inflammatory responses (Nørgård and Svenningsen, 2023). Flavonoid metabolite of apigenin had been proven to have a renoprotective effect in IRI. Apigenin protected renal cells from inflammatory injury due to IRI by inhibiting NF-κB pathway activation via upregulating miR-140-5p and downregulating CXCL12 (He et al., 2021). Formononetin may potently reduce the levels of monocyte chemoattractant protein-1 (MCP-1), IL-1β, and TNF-α, as well as macrophage infiltration in mice with IRI-induced AKI by inhibiting Kruppel-like factor 6 (KLF6)/STAT-mediated proinflammatory polarization of macrophages, thereby significantly alleviating the inflammatory response (Zhang N. X. et al., 2024). In mice with unilateral nephrectomy and renal IRI, naringin exerted renoprotective effects by reducing the expression of IL-1β (Danis et al., 2024). In HK-2 cells after hypoxia/reoxygenation, calycosin exerted a renoprotective effect in IRI by upregulating peroxisome proliferator-activated receptor-gamma (PPARγ), down-regulating early growth response protein 1 (EGR1), and inhibiting inflammatory response mediated by NF-κB (Zhang N. et al., 2022). Kaempferol decreased the levels of vascular cell adhesion molecule 1 (VCAM-1), MCP-1 and ICAM-1, consequently blocking the infiltration of F4/80+ macrophages in sepsis-associated AKI (Xu et al., 2023). Mangiferin promoted Nrf2 expression and inhibited NLRP3 inflammasome activation, thereby reducing IL-1β and IL-18 levels in the CLAP model (He et al., 2014). Chlorogenic acid could effectively inhibit the IL-1β, NLRP3 and caspase-1 levels in renal fibrosis models (Jiao et al., 2024). It had been reported that isoliquiritigenin may alleviate kidney inflammation through suppressing the Mincle/Syk/NF-κB pathway (Liao et al., 2020). Luteolin could prevent the expression of NF-κB, TNF-α and IL-1β, and inhibit MTX-induced nephrotoxicity (Dar et al., 2021). Treatment with luteolin could prevent ADR-induced renal inflammatory damage by elevating IL-10 levels while diminishing TNF-α and IL-1β levels (Owumi et al., 2021). In addition, wogonin suppressed levels of TNF-α, IL-1β, and MCP-1 in a model of high glucose-induced renal tubular epithelial cells (Lei et al., 2021).

### 5.6 Anti-fibrosis

Renal fibrosis is the end result of various chronic kidney diseases. The primary characteristics of fibroblast activation include elevated levels of alpha-smooth muscle actin (α-SMA) expression and substantial production of extracellular matrix (ECM) components (Figure 7). One of the primary drivers of EMT is the TGF-β/Smad axis (Zhou et al., 2026). TGF-β-mediated Smad2/3 phosphorylation and nuclear translocation activate pro-fibrotic transcriptional programs, resulting in sustained ECM accumulation. The turnover of the ECM is governed by a large class of structurally related zinc-dependent endopeptidases called matrix metalloproteinases (MMPs). Multiple MMPs (including MMP10, MMP9, and MMP2) exhibit upregulated expression across a variety of CKDs and can catalyze the degradation of all categories of ECM proteins. Various non-ECM substrates (e.g., E-cadherin) can be cleaved by MMPs (Li L. et al., 2022).

Astragaloside I may alleviate renal fibrosis in DN by regulating the Klotho/transforming growth factor-beta 1 (TGF-β1)/Smad2/3 pathway through simultaneous inhibition of histone deacetylase 3 (HDAC3) and of TGF-β1 (Zhang X. et al., 2024). Apigenin could exert beneficial effects on hypertension-induced renal fibrosis through the transient receptor potential vanilloid 4 (TRPV4)-mediated activation of AMPK/sirtuin 1 (SIRT1) and inhibition of the TGF-β1/Smad2/3 signaling pathway (Wei et al., 2017). Hypertension and CKD are intrinsically related, as hypertension is a significant determinant of the deterioration of renal and cardiovascular outcomes, and a decline in renal function will aggravate hypertension (Burnier and Damianaki, 2023). Kaempferol could reduce the α-SMA, TGF-β1, as well as ECM components in the kidneys of hypertensive rats, consequently alleviating the progressive pathological development of hypertensive renal fibrosis (Guan et al., 2023). Isoliquiritigenin alleviated renal fibrosis via decreasing the fibrosis markers of α-SMA and collagen-III (Liao et al., 2020). Wogonin exerted beneficial effects in diabetic mice renal fibrosis via suppressing the production of collagen-I, fibronectin, and α-SMA, and increasing the production of E-cadherin (Lei et al., 2021). The primary mechanism by which the active metabolites in YPFP interfere with the EMT process is through the inhibition of TGF-β/Smad signaling, thereby alleviating renal fibrosis.

### 5.7 Other

AR could ameliorate disordered lipid metabolism by modulating carnitine palmitoyltransferase 1 (CPT1) expression and reducing ROS and mitochondrial membrane potential loss (Li A. P. et al., 2020). KEGG pathway analysis indicated that regulating the gonadotropin-releasing hormone (GnRH) and vascular endothelial growth factor (VEGF) pathway was the key pathways in AR saponins’ treatment of ADR-induced nephropathy (Li et al., 2023). Genistein is a natural bioactive flavonoid with broad pharmacological activities, applicable for treating various diseases, particularly chronic metabolic diseases (Peng et al., 2022). Methylglyoxal (MGO), as a key precursor of advanced glycation end products, cycles at elevated concentrations in the blood and plays a significant role in the diabetic disease process. In type 1 and type 2 diabetes models, genistein inhibited the generation of advanced glycation end products by capturing MGO to form adducts and increasing the levels of aldose reductase, as well as glyoxalase I and II, in the kidneys (Zhao et al., 2019). Mitochondrial homeostasis was maintained by oroxylin A through activation of the PPARα-BNIP3 pathway (Yao et al., 2022). Neochlorogenic acid exhibited significant potential protective effects on glucolipotoxicity-induced DN by regulating NF-κB pathway (Hung et al., 2023). Esculetin had shown promising renoprotection by upregulating the PINK1/Parkin-mediated mitophagy, as evidenced by increasing PINK1, Parkin, LC3B, and decreasing p62 expression (Dagar et al., 2025).

## 6 Safety of YPFP

A study analyzed the adverse reactions associated with YPF-containing formulations used to treat upper respiratory tract infections, rhinitis, and the common cold, collecting 158 clinical reports (Fan et al., 2026). Adverse reactions typically occur 1 to 3 days after taking the medication and primarily affect the gastrointestinal system, the skin and subcutaneous tissues, and the nervous system. In clinical reports on the use of YPF for the treatment of kidney disease, a small number of patients experienced adverse reactions, such as nausea, lung infection, coagulation disorders, infection, and gastrointestinal bleeding (Wang et al., 2020; Liu et al., 2021; Li, 2025) (Table 3). Although YPFP has demonstrated significant clinical efficacy in treating kidney disease, there is an equally urgent need for more well-designed, larger-scale trials to evaluate the efficacy and safety of this important herbal formula.

**TABLE 3:** Adverse reactions of YPFP on renal diseases.

Disease type	Dosage forms	Dose and course of treatment	Combined medication	YPF/Control (n)	Result	Side effects	Refs
Nephrotic syndrome	YPFP	24 g/d, t.i.d., 28 days	Rituximab injection (28 days)	46/46	BUN↓, Scr↓, Serum Albumin↑	Nausea, lung infection, and coagulation disorders	Li (2025)
Primary nephrotic syndrome	YPF granules	15 g/d, t.i.d., 9 months	Tacrolimus (0.1 mg/kg·d, b.i.d., 9 months)	48/49	BUN↓, Scr↓, CD4^+^/CD8^+^↑, IgG↑, IgA↑	Gastrointestinal discomfort	Wang et al. (2020)
Primary nephrotic syndrome	YPF granules	15 g/d, t.i.d, 9 months	Tacrolimus capsule (0.1 mg/kg·d, b.i.d, 6 months)	50/50	BUN↓, Scr↓, IgG↑, IgA↑, IgM↑, TGF-β1↓, IL-2↓, IL-10↑, IL-6↓	Nausea, infection, and gastrointestinal bleeding	Liu et al. (2021)

YPF, the YPF-treated group; Control, the control group.

## 7 Conclusions and discussion

To date, YPFP, its botanical drugs, and bioactive metabolites have shown significant roles in the prophylaxis and therapeutic management of renal diseases. Its mechanisms of action involve multiple molecular processes, including anti-apoptosis, immunoregulation, anti-OS, anti-ferroptosis, and anti-inflammation. Among these complex pathological mechanisms, inflammation, mitophagy and ferroptosis are involved in the transformation from AKI to CKD. Numerous studies utilizing genetically modified animals of the Nrf2 system have demonstrated ameliorative effects of Nrf2 against AKI-to-CKD progression (Ng et al., 2024). Moreover, downregulation of HDAC3 can promote GPX-4 expression, enhance antioxidant defense, and prevent AKI-to-CKD transition (Zhou et al., 2026). During AKI, upregulation of autophagy alleviates oxidative stress and renal fibrosis, as exemplified by α-Klotho, which safeguards renal cells and delays the AKI-to-CKD transition by enhancing autophagic flux. Inflammatory responses and mitochondrial dysfunction represent key mechanisms driving the AKI-to-CKD transition. The mitophagy-NLRP3 inflammasome axis interacts during AKI-CKD transition, co-regulating renal injury progression and repair. Pathways including PINK1/PGC-1α, BNIP3/HIF-1α, and mTOR participate in this process with intricate mechanisms (Zhu et al., 2025). The active metabolites in YPFP may be involved in the above process, delaying the transformation from AKI to CKD.

These studies also have some shortcomings, for example, the straightforward binding motif between liquiritigenin and VKORC1 has not been confirmed, nor has the existence of other downstream pathways been established (Guo et al., 2024). In addition, although many *in vivo* models have demonstrated the mechanism of action of YPFP, further validation using inhibitors or knockout mice will still be required in the future.

In recent years, the “gut-kidney axis” has garnered widespread attention as a key pathway for immune regulation (Miao et al., 2025). Research has found that Jiawei YPF polysaccharides enhance immune function and maintain gut health in mice (Wu et al., 2025). At the same time, numerous studies have confirmed that YPF polysaccharides can improve the gut microbiota of chickens (Guan et al., 2024). Research has shown that AR aqueous extract modulates gut microbiota disorders caused by kidney disease by reducing the relative abundance of the Firmicutes and increasing that of Bacteroidetes (Qu et al., 2025). Therefore, it is hypothesized that YPFP and its active metabolites may exert a renoprotective effect by modulating the gut microbiota. High-quality data from *in vivo* animal studies and clinical trials are still needed to validate this hypothesis.

In addition, IgA nephropathy (IgAN) is also a major cause of ESRD. Mucosal immunity plays a significant role in the pathogenesis of IgAN, in which abnormal immunoglobulin A1 (IgA1) molecules produced by gut-associated lymphoid tissue enter the circulation and subsequently deposit in the kidneys (Liu T. et al., 2025). This deposition triggers a series of local inflammatory responses mediated by mesangial cells, leading to glomerular damage and interstitial fibrosis. Galactose-deficient IgA1 (Gd-IgA1) is an abnormally glycosylated form of IgA1 that plays a central role in the pathogenesis of IgAN. Inhibition of Nrf2 reversed the suppressive effects of luteolin, an active metabolite in YPFP, on Gd-IgA1-induced mesangial cell inflammation, oxidative stress, and ECM protein expression (Liang et al., 2025). In Gd-IgA1-induced HBZY-1 cells treated with 10 μM luteolin, inhibition of Nrf2 expression led to increased levels of TNF-α, IL-1, and IL-6. The expression of proteins related to ECM accumulation, including TGF-β1, and type IV collagen (Col IV), was increased due to Nrf2 inactivation.

Overall, YPFP is a promising candidate drug for the treatment of kidney diseases. Its bioactive metabolites, particularly flavonoid and polysaccharide, hold promise for development and application as active metabolites. However, it is known that flavonoids have poor bioavailability when taken orally. Further research is needed to improve their bioavailability for clinical development and application.

## References

[B1] Agrawal S. Zaritsky J. J. Fornoni A. Smoyer W. E. (2018). Dyslipidaemia in nephrotic syndrome: mechanisms and treatment. Nat. Reviews. Nephrol. 14 (1), 57–70. 10.1038/nrneph.2017.155 29176657 PMC5770189

[B2] Alam M. A. Sernia C. Brown L. (2013). Ferulic acid improves cardiovascular and kidney structure and function in hypertensive rats. J. Cardiovascular Pharmacology 61 (3), 240–249. 10.1097/FJC.0b013e31827cb600 23188120

[B3] Amini N. Shoshtari M. H. Nejaddehbashi F. Dianat M. Badavi M. (2024). Dose-dependent renoprotective effect of vanillic acid on methotrexate-induced nephrotoxicity via its anti-apoptosis, antioxidant, and anti-inflammatory properties. Naunyn-Schmiedeberg's Archives Pharmacology 397 (6), 4195–4204. 10.1007/s00210-023-02866-y 38041776

[B5] Bai Y. Wei W. Yao C. Wu S. Wang W. Guo D. A. (2023). Advances in the chemical constituents, pharmacological properties and clinical applications of TCM formula Yupingfeng san. Fitoterapia 164, 105385. 10.1016/j.fitote.2022.105385 36473539

[B6] Burnier M. Damianaki A. (2023). Hypertension as cardiovascular risk factor in chronic kidney disease. Circulation Research 132 (8), 1050–1063. 10.1161/CIRCRESAHA.122.321762 37053276

[B7] Caglayan C. Kandemir F. M. Darendelioğlu E. Küçükler S. Ayna A. (2021). Hesperidin protects liver and kidney against sodium fluoride-induced toxicity through anti-apoptotic and anti-autophagic mechanisms. Life Sciences 281, 119730. 10.1016/j.lfs.2021.119730 34147482

[B8] Chen Q. He H. Li P. Zhu J. Xiong M. (2013). Identification and quantification of atractylenolide I and atractylenolide III in rhizoma Atractylodes macrocephala by liquid chromatography-ion trap mass spectrometry. Biomed. Chromatography BMC 27 (6), 699–707. 10.1002/bmc.2847 23175447

[B9] Chen D. Guo Y. Li P. (2024). New insights into a novel metabolic biomarker and therapeutic target for chronic kidney disease. Integr. Med. Nephrol. Androl. 11 (3), e24–00019. 10.1097/IMNA-D-24-00019

[B10] Chen L. Deng Y. Hu J. Gong X. (2025). Nephroprotective effects of substances of medicine food homology and traditional Chinese medicine phytochemicals against acute kidney injury. Front. Pharmacology 16, 1539886. 10.3389/fphar.2025.1539886 PMC11880292 40046749

[B11] Costantino V. V. Gil Lorenzo A. F. Bocanegra V. Vallés P. G. (2021). Molecular mechanisms of hypertensive nephropathy: renoprotective effect of losartan through Hsp70. Cells 10 (11), 3146. 10.3390/cells10113146 34831368 PMC8619557

[B12] Dagar N. Habshi T. Shelke V. Jadhav H. R. Gaikwad A. B. (2025). Esculetin and phloretin combination mitigates acute kidney injury-diabetes comorbidity via regulating mitophagy and inflammation: a dual-pronged approach. Phytotherapy Research PTR 39, 2533–2554. 10.1002/ptr.8489 40159308

[B13] Danis E. G. Acar G. Dasdelen D. Solmaz M. Mogulkoc R. Baltaci A. K. (2024). Naringin affects Caspase-3, IL-1β, and HIF-1α levels in experimental kidney ischemia-reperfusion in rats. Curr. Pharmaceutical Design 30 (42), 3339–3349. 10.2174/0113816128324562240816095551 39229980

[B14] Dar A. A. Fehaid A. Alkhatani S. Alarifi S. Alqahtani W. S. Albasher G. (2021). The protective role of luteolin against the methotrexate-induced hepato-renal toxicity via its antioxidative, anti-inflammatory, and anti-apoptotic effects in rats. Hum. and Experimental Toxicology 40 (7), 1194–1207. 10.1177/0960327121991905 33530773

[B15] Du C. Y. Choi R. C. Zheng K. Y. Dong T. T. Lau D. T. Tsim K. W. (2013). Yu ping feng san, an ancient Chinese herbal decoction containing astragali radix, atractylodis macrocephalae rhizoma and saposhnikoviae radix, regulates the release of cytokines in murine macrophages. PloS One 8 (11), e78622. 10.1371/journal.pone.0078622 24244327 PMC3823765

[B16] Fan J. Li Y. Liu H. Li X. Liu X. (2026). “Analysis of quality control and adverse reaction reports for 158 cases of Yupingfeng preparations,” in China Brand and Anti-counterfeiting (5), 43–45.

[B17] Fang C. Y. Lou D. Y. Zhou L. Q. Wang J. C. Yang B. He Q. J. (2021). Natural products: potential treatments for cisplatin-induced nephrotoxicity. Acta Pharmacologica Sin. 42 (12), 1951–1969. 10.1038/s41401-021-00620-9 PMC8633358 33750909

[B18] Flores-Morales V. Villasana-Ruíz A. P. Garza-Veloz I. González-Delgado S. Martinez-Fierro M. L. (2023). Therapeutic effects of coumarins with different substitution patterns. Mol. Basel, Switz. 28 (5), 2413. 10.3390/molecules28052413 PMC10005689 36903660

[B19] Gao J. Li J. Shao X. Jin Y. Lü X. W. Ge J. F. (2009). Antiinflammatory and immunoregulatory effects of total glucosides of Yupingfeng powder. Chin. Medical Journal 122 (14), 1636–1641. 10.3760/cma.j.issn.0366-6999.2009.14.007 19719964

[B20] Geng X. Zhong D. Su L. Lin Z. Yang B. (2020). Preventive and therapeutic effect of Ganoderma lucidum on kidney injuries and diseases. Adv. Pharmacology (San Diego, Calif.) 87, 257–276. 10.1016/bs.apha.2019.10.003 32089235

[B21] Girndt M. (2017). Diagnosis and treatment of chronic kidney disease. Der Internist 58 (3), 243–256. 10.1007/s00108-017-0195-2 28194476

[B22] Guan Y. Quan D. Chen K. Kang L. Yang D. Wu H. (2023). Kaempferol inhibits renal fibrosis by suppression of the sonic hedgehog signaling pathway. Phytomedicine International Journal Phytotherapy Phytopharmacology 108, 154246. 10.1016/j.phymed.2022.154246 36274411

[B23] Guan Y. Zheng W. Bai Y. Wu B. (2024). Yupingfeng polysaccharide promote the growth of chickens via regulating gut microbiota. Front. Veterinary Science 11, 1337698. 10.3389/fvets.2024.1337698 PMC10920335 38464700

[B24] Guo S. Li J. Feng S. Yu S. Wang K. (2002). The effect of jiaweiyupingfeng decoction on the MDA and the XOD in blood plasma of the white mouse with kidney yang deficiency. J. Qingdao Agric. Univ. Nat. Sci. 19 (2), 151–152+160. 10.3969/j.issn.1674-148X.2002.02.019

[B25] Guo R. Z. Li J. Pan S. K. Hu M. Y. Lv L. X. Feng Q. (2024). Liquiritigenin, an active ingredient of liquorice, alleviates acute kidney injury by VKORC1-Mediated ferroptosis inhibition. Am. Journal Chin. Medicine 52 (5), 1507–1526. 10.1142/S0192415X24500599 39192677

[B26] Guo Z. Y. Wu X. Zhang S. J. Yang J. H. Miao H. Zhao Y. Y. (2025). Poria cocos: traditional uses, triterpenoid components and their renoprotective pharmacology. Acta Pharmacol. Sin. 46 (4), 836–851. 10.1038/s41401-024-01404-7 39482471 PMC11950336

[B28] Hayama N. (2003). Chronic glomerulonephritis syndrome (CGNS). J. Nippon Med. Sch. = Nippon Ika Daigaku Zasshi 70 (4), 360–362. 10.1272/jnms.70.360 12928718

[B29] He L. Peng X. Zhu J. Chen X. Liu H. Tang C. (2014). Mangiferin attenuate sepsis-induced acute kidney injury via antioxidant and anti-inflammatory effects. Am. Journal Nephrology 40 (5), 441–450. 10.1159/000369220 25427663

[B30] He X. Wen Y. Wang Q. Wang Y. Zhang G. Wu J. (2021). Apigenin nanoparticle attenuates renal ischemia/reperfusion inflammatory injury by regulation of miR-140-5p/CXCL12/NF-κB signaling pathway. J. Biomedical Nanotechnology 17 (1), 64–77. 10.1166/jbn.2021.3010 33653497

[B31] He Q. Huang M. Wang T. Gong L. Ma Z. Ye F. (2025). The effect of Yupingfeng polysaccharides on immune performance and intestinal microbiota in goslings. Animals 15 (14), 2077. 10.3390/ani15142077 40723539 PMC12291793

[B32] He Z. Li M. Chen H. Jiang R. Lin J. Cai Y. (2025). The renoprotective effects of Radix astragali and Salvia miltiorrhiza bunge on short-term outcomes of acute kidney injury: a retrospective study. Integr. Med. Nephrol. Androl. 12 (1), e24–e42. 10.1097/IMNA-D-24-00042

[B33] Hu Q. Qu C. Xiao X. Zhang W. Jiang Y. Wu Z. (2021). Flavonoids on diabetic nephropathy: advances and therapeutic opportunities. Chin. Medicine 16 (1), 74. 10.1186/s13020-021-00485-4 PMC8349014 34364389

[B34] Huang L. Y. Chen D. F. Wu T. Gao Y. J. (2024). Simultaneous quantitation of 15 bioactive components in Yupingfeng granules by LC-MS/MS. J. Mass Spectrometry JMS 59 (5), e5024. 10.1002/jms.5024 38605459

[B35] Huang L. Liu M. Shen L. Chen D. Wu T. Gao Y. (2025). Polysaccharides from Yupingfeng granules ameliorated cyclophosphamide-induced immune injury by protecting intestinal barrier. Int. Immunopharmacology 146, 113866. 10.1016/j.intimp.2024.113866 39709910

[B36] Hung T. W. Yang M. Y. Yu M. H. Tsai I. N. Tsai Y. C. Chan K. C. (2023). Mulberry leaf extract and neochlorogenic acid ameliorate glucolipotoxicity-induced diabetic nephropathy in high-fat diet-fed db/db mice. Food and Function 14 (19), 8975–8986. 10.1039/d3fo02640j 37732507

[B37] Jiang P. Yao C. Guo D. A. (2024). Traditional Chinese medicine for the treatment of immune-related nephropathy: a review. Acta Pharmaceutica Sin. B 14 (1), 38–66. 10.1016/j.apsb.2023.11.006 PMC10793104 38239236

[B38] Jiao H. Zhang M. Xu W. Pan T. Luan J. Zhao Y. (2024). Chlorogenic acid alleviate kidney fibrosis through regulating TLR4/NF-қB mediated oxidative stress and inflammation. J. Ethnopharmacology 335, 118693. 10.1016/j.jep.2024.118693 39142620

[B39] Kang K. P. Park S. K. Kim D. H. Sung M. J. Jung Y. J. Lee A. S. (2011). Luteolin ameliorates cisplatin-induced acute kidney injury in mice by regulation of p53-dependent renal tubular apoptosis. Nephrol. Dialysis, Transplantation 26 (3), 814–822. 10.1093/ndt/gfq528 20817674

[B40] Karacaer C. Erdogan D. G. Tanyeli A. Baylan H. Eraslan E. Guler M. C. (2023). The protective effects of hesperidin pretreatment on kidney and remote organs against renal ischemia-reperfusion injury. Eur. Review Medical Pharmacological Sciences 27 (7), 2808–2814. 10.26355/eurrev_202304_31911 37070880

[B41] Kassab R. B. Elhenawy A. A. AbdulrahmanTheyab Hawsawi Y. M. Al-Amer O. M. Oyouni A. A. A. (2023). Modulation of inflammatory, oxidative, and apoptotic stresses mediates the renoprotective effect of daidzein against glycerol-induced acute kidney injury in rats. Environ. Sci. Pollut. Res. Int. 30 (56), 119016–119033. 10.1007/s11356-023-30461-4 37919499

[B42] Khalaf M. M. Hassan S. M. Sayed A. M. Abo-Youssef A. M. (2022). Ameliorate impacts of scopoletin against vancomycin-induced intoxication in rat model through modulation of Keap1-Nrf2/HO-1 and IκBα-P65 NF-κB/P38 MAPK signaling pathways: molecular study, molecular docking evidence and network pharmacology analysis. Int. Immunopharmacology 102, 108382. 10.1016/j.intimp.2021.108382 34848155

[B43] Khatun J. Gelles J. D. Chipuk J. E. (2024). Dynamic death decisions: how mitochondrial dynamics shape cellular commitment to apoptosis and ferroptosis. Dev. Cell 59 (19), 2549–2565. 10.1016/j.devcel.2024.09.004 39378840 PMC11469553

[B44] Kim M. K. Yang D. H. Jung M. Jung E. H. Eom H. Y. Suh J. H. (2011). Simultaneous determination of chromones and coumarins in Radix saposhnikoviae by high performance liquid chromatography with diode array and tandem mass detectors. J. Chromatography. A 1218 (37), 6319–6330. 10.1016/j.chroma.2011.06.103 21807369

[B45] Kundu S. Ghosh S. Sahu B. D. (2024). Scopoletin alleviates high glucose-induced toxicity in human renal proximal tubular cells via inhibition of oxidative damage, epithelial-mesenchymal transition, and fibrogenesis. Mol. Biology Reports 51 (1), 620. 10.1007/s11033-024-09579-2 38709349

[B46] Lan T. Y. Dun R. L. Yao D. S. Wu F. Qian Y. L. Zhou Y. (2023). Effects of resveratrol on renal ischemia-reperfusion injury: a systematic review and meta-analysis. Front. Nutrition 9, 1064507. 10.3389/fnut.2022.1064507 PMC9845714 36687723

[B47] Lee K. Jang H. R. Rabb H. (2024). Lymphocytes and innate immune cells in acute kidney injury and repair. Nat. Reviews. Nephrol. 20 (12), 789–805. 10.1038/s41581-024-00875-5 39095505

[B48] Lei L. Zhao J. Liu X. Q. Chen J. Qi X. M. Xia L. L. (2021). Wogonin alleviates kidney tubular epithelial injury in diabetic nephropathy by inhibiting PI3K/Akt/NF-κB signaling pathways. Drug Design, Development Therapy 15, 3131–3150. 10.2147/DDDT.S310882 34295152 PMC8291679

[B49] Li D. (2025). A study on the efficacy of Yupingfeng san combined with rituximab in the treatment of children with refractory nephrotic syndrome. J. Med. Theory Pract. 38 (06), 1001–1003. 10.19381/j.issn.1001-7585.2025.06.032

[B50] Li C. Y. Song H. T. Wang X. X. Wan Y. Y. Ding X. S. Liu S. J. (2017). Urinary metabolomics reveals the therapeutic effect of HuangQi injections in cisplatin-induced nephrotoxic rats. Sci. Reports 7 (1), 3619. 10.1038/s41598-017-03249-z 28620200 PMC5472607

[B51] Li A. P. Yang L. Cui T. Zhang L. C. Liu Y. T. Yan Y. (2020). Uncovering the mechanism of astragali radix against nephrotic syndrome by intergrating lipidomics and network pharmacology. Phytomedicine International Journal Phytotherapy Phytopharmacology 77, 153274. 10.1016/j.phymed.2020.153274 32771537

[B52] Li J. Cao F. Yin H. L. Huang Z. J. Lin Z. T. Mao N. (2020). Ferroptosis: past, present and future. Cell Death and Disease 11 (2), 88. 10.1038/s41419-020-2298-2 32015325 PMC6997353

[B53] Li M. Gao Y. Yue X. Zhang B. Zhou H. Yuan C. (2021). Integrated metabolomics and network pharmacology approach to reveal immunomodulatory mechanisms of Yupingfeng granules. J. Pharmaceutical Biomedical Analysis 194, 113660. 10.1016/j.jpba.2020.113660 33261954

[B54] Li L. Fu H. Liu Y. (2022). The fibrogenic niche in kidney fibrosis: components and mechanisms. Nat. Reviews. Nephrol. 18 (9), 545–557. 10.1038/s41581-022-00590-z 35788561

[B55] Li Y. Liu Y. Sun Y. Ma S. Ma C. Zhou H. (2022). Study on the mechanism of Yupingfeng powder in the treatment of immunosuppression based on UPLC⁃QTOF⁃MS, network pharmacology and molecular biology verification. Life Sciences 289, 120211. 10.1016/j.lfs.2021.120211 34875251

[B56] Li A. Cui W. Zhao Y. Luo T. Zhang Q. Liu Y. (2023). Exploration of the main effective constituent and the mechanism in astragali radix in the treatment for doxorubicin-induced nephropathy by integrating metabolomics and molecular docking. J. Ethnopharmacology 305, 116074. 10.1016/j.jep.2022.116074 36577490

[B57] Li X. J. Suo P. Wang Y. N. Zou L. Nie X. L. Zhao Y. Y. (2024). Arachidonic acid metabolism as a therapeutic target in AKI-to-CKD transition. Front. Pharmacol. 15, 1365802. 10.3389/fphar.2024.1365802 38523633 PMC10957658

[B58] Li L. Cai W. Zhang H. Tang J. Yang Y. Huang Y. (2025). Bergapten ameliorates renal fibrosis by inhibiting ferroptosis. Phytotherapy Research PTR 39 (3), 1355–1371. 10.1002/ptr.8425 39764683

[B59] Li X. Wang B. Guan D. Zhou J. Men Z. Sun Y. (2025a). A research method of compatibility mechanism of traditional Chinese medicine prescription-taking Yupingfeng san as an example. J. Ethnopharmacol. 348, 119815. 10.1016/j.jep.2025.119815 40268108

[B60] Li X. Zhao C. Qi J. (2025b). Identification of active markers of Chinese formula Yupingfeng san by network pharmacology and HPLC-Q-TOF-MS/MS analysis in experimental allergic rhinitis models of mice and isolated basophilic leukemia cell line RBL-2H3. Pharm. Basel, Switz. 18 (4), 540. 10.3390/ph18040540 PMC12030416 40283975

[B61] Li S. Jing S. Zhou J. Li H. Yin P. Su L. (2026). Mechanisms of traditional Chinese medicine in regulating Nrf2-related signaling pathways for the treatment of oligoasthenozoospermia: a review. J. Ethnopharmacol. 360, 121162. 10.1016/j.jep.2026.121162 41485624

[B62] Liang D. Y. Cong S. H. Li L. H. Yi Q. Q. Tang L. P. Cao L. O. (2025). Luteolin delays the progression of IgA nephropathy by attenuating inflammation, oxidative stress and reducing extracellular matrix accumulation through activating the Nrf-2/HO-1 pathway. Front. Pharmacology 16, 1530655. 10.3389/fphar.2025.1530655 PMC12279811 40697655

[B63] Liao Y. Tan R. Z. Li J. C. Liu T. T. Zhong X. Yan Y. (2020). Isoliquiritigenin attenuates UUO-induced renal inflammation and fibrosis by inhibiting Mincle/Syk/NF-Kappa B signaling pathway. Drug Design, Development Therapy 14, 1455–1468. 10.2147/DDDT.S243420 32341639 PMC7166058

[B64] Liu Y. Cao H. Wang X. Sun S. Tian K. Chang H. (2021). Effect of Yupingfeng granule combined with tacrolimus capsule on renal function, humoral immune function and Th1/Th2 cell balance in children with primary nephrotic syndrome. Prog. Mod. Biomed. 21 (01), 184–187+192. 10.13241/j.cnki.pmb.2021.01.041

[B65] Liu K. Yu M. He Y. Wang T. Li G. Wang L. (2025). Inhibitory effects of herbal monomers on ferroptosis in renal fibrosis: a review and mechanistic study. Front. Pharmacology 16, 1610573. 10.3389/fphar.2025.1610573 PMC12321836 40766752

[B66] Liu T. Zhuang X. X. Zheng W. J. Gao J. R. (2025). Integrative multi-omics and network pharmacology reveal the mechanisms of fangji huangqi decoction in treating IgA nephropathy. J. Ethnopharmacol 337 (Pt 3), 118996. 10.1016/j.jep.2024.118996 39490710

[B67] Losappio V. Franzin R. Infante B. Godeas G. Gesualdo L. Fersini A. (2020). Molecular mechanisms of premature aging in hemodialysis: the complex interplay between innate and adaptive immune dysfunction. Int. Journal Molecular Sciences 21 (10), 3422. 10.3390/ijms21103422 32408613 PMC7279398

[B68] Ma X. Tu P. Chen Y. Zhang T. Wei Y. Ito Y. (2004). Preparative isolation and purification of isoflavan and pterocarpan glycosides from Astragalus membranaceus bge. Var. mongholicus (Bge.) hsiao by high-speed counter-current chromatography. J. Chromatography. A 1032 (2), 311–315. 10.1016/j.chroma.2003.10.014 14753698

[B69] Ma Q. Xu Y. Tang L. Yang X. Chen Z. Wei Y. (2020). Astragalus polysaccharide attenuates cisplatin-induced acute kidney injury by suppressing oxidative damage and mitochondrial dysfunction. BioMed Research International 2020, 2851349. 10.1155/2020/2851349 31998784 PMC6970487

[B70] Ma L. Feng Z. Li P. (2024). New insights into the use of traditional Chinese medicine for treating diabetic kidney disease by regulating DNA methylation. Integr. Med. Nephrol. Androl. 11 (3), e24–00018. 10.1097/imna-d-24-00018

[B71] Ma X. Guan B. Pang L. (2025). Calycosin ameliorates albuminuria in nephrotic syndrome by targeting Notch1/Snail pathway. BMC Nephrology 26 (1), 198. 10.1186/s12882-025-04113-3 40251522 PMC12008911

[B72] Maremonti F. Meyer C. Linkermann A. (2022). Mechanisms and models of kidney tubular necrosis and nephron loss. J. Am. Soc. Nephrol. JASN 33 (3), 472–486. 10.1681/ASN.2021101293 35022311 PMC8975069

[B73] Martina M. N. Noel S. Bandapalle S. Hamad A. R. Rabb H. (2014). T lymphocytes and acute kidney injury: update. Nephron. Clin. Practice 127 (1-4), 51–55. 10.1159/000363719 25343821 PMC5264523

[B74] Meng H. Yang Q. Liu C. Tan Y. (2015). Clinical observation on the treatment of modified yuping fengsan proteinuria of chronic glomerulonephritis. Mod. Chin. Med. 35 (1), 8–10. 10.13424/j.cnki.mtcm.2015.01.004

[B75] Miao H. Zhang S. J. Wu X. Li P. Zhao Y. Y. (2025). Tryptophan metabolism as a target in gut microbiota, ageing and kidney disease. Int. Journal Biological Sciences 21 (10), 4374–4387. 10.7150/ijbs.115359 PMC12320236 40765836

[B76] Ng C. Kim M. Kwak M. K. (2024). Oxidative stress and NRF2 signaling in kidney injury. Toxicol. Research 41 (2), 131–147. 10.1007/s43188-024-00272-x PMC11850685 40013079

[B77] Nikles S. Monschein M. Zou H. Liu Y. He X. Fan D. (2017). Metabolic profiling of the traditional Chinese medicine formulation Yu ping feng san for the identification of constituents relevant for effects on expression of TNF-α, IFN-γ, IL-1β and IL-4 in U937 cells. J. Pharmaceutical Biomedical Analysis 145, 219–229. 10.1016/j.jpba.2017.03.049 28667937

[B78] Nørgård M. Ø. Svenningsen P. (2023). Acute kidney injury by ischemia/reperfusion and extracellular vesicles. Int. J. Mol. Sci. 24 (20), 15312. 10.3390/ijms242015312 37894994 PMC10607034

[B79] Nowak K. L. Edelstein C. L. (2020). Apoptosis and autophagy in polycystic kidney disease (PKD). Cell. Signalling 68, 109518. 10.1016/j.cellsig.2019.109518 31881325 PMC7127985

[B80] Østergaard J. A. Cooper M. E. Jandeleit-Dahm K. A. M. (2020). Targeting oxidative stress and anti-oxidant defence in diabetic kidney disease. J. Nephrology 33 (5), 917–929. 10.1007/s40620-020-00749-6 32447617

[B81] Owumi S. E. Lewu D. O. Arunsi U. O. Oyelere A. K. (2021). Luteolin attenuates doxorubicin-induced derangements of liver and kidney by reducing oxidative and inflammatory stress to suppress apoptosis. Hum. and Experimental Toxicology 40 (10), 1656–1672. 10.1177/09603271211006171 33827303

[B82] Oza M. J. Kulkarni Y. A. (2019). Formononetin attenuates kidney damage in type 2 diabetic rats. Life Sciences 219, 109–121. 10.1016/j.lfs.2019.01.013 30641085

[B83] Pei T. Dai Y. Tan X. Geng A. Li S. Gui Y. (2023). Yupingfeng san exhibits anticancer effect in hepatocellular carcinoma cells via the MAPK pathway revealed by HTS2 technology. J. Ethnopharmacology 306, 116134. 10.1016/j.jep.2023.116134 36627003

[B84] Peng Q. Li Y. Shang J. Huang H. Zhang Y. Ding Y. (2022). Effects of genistein on common kidney diseases. Nutrients 14 (18), 3768. 10.3390/nu14183768 36145144 PMC9506319

[B85] Perazella M. A. Rosner M. H. (2022). Drug-induced acute kidney injury. Clin. Journal Am. Soc. Nephrol. CJASN 17 (8), 1220–1233. 10.2215/CJN.11290821 35273009 PMC9435983

[B86] Privratsky J. R. Ide S. Chen Y. Kitai H. Ren J. Fradin H. (2023). A macrophage-endothelial immunoregulatory axis ameliorates septic acute kidney injury. Kidney International 103 (3), 514–528. 10.1016/j.kint.2022.10.008 36334787 PMC9974788

[B87] Qu L. Jiao B. (2023). The interplay between immune and metabolic pathways in kidney disease. Cells 12 (12), 1584. 10.3390/cells12121584 37371054 PMC10296595

[B88] Qu T. Zhang N. Cai J. Li C. Zhang Y. Xu X. (2025). Integrated bioinformatics and multi-omics reveal astragalus extract's gut-kidney axis mechanism in hyperuricemic nephropathy via purine metabolism. Phytomedicine 146, 157121. 10.1016/j.phymed.2025.157121 40774007

[B89] Ramazanoglu M. A. Toprak T. Erdem M. R. Gumrukcu G. Kucuk H. Sengor F. (2020). Effects of butein on renal ischemia/reperfusion injury: an experimental study. Arch. Ital. Urol. Androl. organo Uff. Soc. Ital. Ecogr. Urol. Nefrol. 92 (4), 335–339. 10.4081/aiua.2020.4.335 33348962

[B90] Sahindokuyucu-Kocasari F. Akyol Y. Ozmen O. Erdemli-Kose S. B. Garli S. (2021). Apigenin alleviates methotrexate-induced liver and kidney injury in mice. Hum. and Experimental Toxicology 40 (10), 1721–1731. 10.1177/09603271211009964 33845614

[B91] Sai Y. P. Song Y. C. Chen X. X. Luo X. Liu J. Cui W. J. (2018). Protective effect of astragalosides from Radix astragali on adriamycin-induced podocyte injury. Exp. Therapeutic Medicine 15 (5), 4485–4490. 10.3892/etm.2018.5933 29731833 PMC5921218

[B92] Sanz A. B. Sanchez-Niño M. D. Ramos A. M. Ortiz A. (2023). Regulated cell death pathways in kidney disease. Nat. Reviews. Nephrol. 19 (5), 281–299. 10.1038/s41581-023-00694-0 PMC10035496 36959481

[B93] Shen Z. L. Zhao L. Bao S. F. (1988). Therapeutic effect and experimental study of yupingfeng powder in the treatment of chronic renal failure with infection. Chin. J. Integr. Traditional West. Med. 8 (5), 268–270. 10.7661/CJIM.1988.5.268 3197228

[B94] Shen Q. Fang J. Guo H. Su X. Zhu B. Yao X. (2023). Astragaloside IV attenuates podocyte apoptosis through ameliorating mitochondrial dysfunction by up-regulated Nrf2-ARE/TFAM signaling in diabetic kidney disease. Free Radical Biology and Medicine 203, 45–57. 10.1016/j.freeradbiomed.2023.03.022 37030337

[B95] Shi X. Zhong X. Ding J. (2018). Adjuvant treatment with Yupingfeng formula for primary nephrotic syndrome in children: a PRISMA systematic review and meta-analysis of randomized controlled trials. Medicine 97 (29), e11598. 10.1097/MD.0000000000011598 30024564 PMC6086467

[B96] Shi Y. Shi X. Zhao M. Ma S. Zhang Y. (2024). Pharmacological potential of astragali radix for the treatment of kidney diseases. Phytomedicine International Journal Phytotherapy Phytopharmacology 123, 155196. 10.1016/j.phymed.2023.155196 37952410

[B97] Singh V. Kaur R. Kumari P. Pasricha C. Singh R. (2023). ICAM-1 and VCAM-1: gatekeepers in various inflammatory and cardiovascular disorders. Clin. Chimica Acta; International Journal Clinical Chemistry 548, 117487. 10.1016/j.cca.2023.117487 37442359

[B98] Sun X. Cui X. B. Wen H. M. Shan C. X. Wang X. Z. Kang A. (2017). Influence of sulfur fumigation on the chemical profiles of Atractylodes macrocephala Koidz. evaluated by UFLC-QTOF-MS combined with multivariate statistical analysis. J. Pharmaceutical Biomedical Analysis 141, 19–31. 10.1016/j.jpba.2017.03.003 28414971

[B99] Sun Z. Kong Y. Li N. Jiang X. Cao T. Jia Y. (2018). Effect of Yupingfeng granules on the skin barrier in atopic dermatitis mice models. J. Traditional Chin. Medicine 38 (6), 872–878. 32186134

[B100] Tang S. Xiao X. (2020). Ferroptosis and kidney diseases. Int. Urology Nephrology 52 (3), 497–503. 10.1007/s11255-019-02335-7 31768804

[B101] Tang L. Liu Y. Wang Y. Long C. (2010). Phytochemical analysis of an antiviral fraction of Radix astragali using HPLC-DAD-ESI-MS/MS. J. Natural Medicines 64 (2), 182–186. 10.1007/s11418-009-0381-1 20037801

[B102] Tang C. Livingston M. J. Safirstein R. Dong Z. (2023). Cisplatin nephrotoxicity: new insights and therapeutic implications. Nat. Reviews. Nephrol. 19 (1), 53–72. 10.1038/s41581-022-00631-7 36229672

[B103] Tao X. Gao J. (2011). Clinical study on YuPingFeng powder to prevent cold and regulate immune balance of the patients with chronic nephritis. J. Sichuan Traditional Chin. Med. 29 (7), 86–89.

[B104] Tomar A. Kaushik S. Khan S. I. Bisht K. Nag T. C. Arya D. S. (2020). The dietary isoflavone daidzein mitigates oxidative stress, apoptosis, and inflammation in CDDP-Induced kidney injury in rats: impact of the MAPK signaling pathway. J. Biochemical Molecular Toxicology 34 (2), e22431. 10.1002/jbt.22431 31833131

[B105] Vargas F. Romecín P. García-Guillén A. I. Wangesteen R. Vargas-Tendero P. Paredes M. D. (2018). Flavonoids in kidney health and disease. Front. Physiology 9, 394. 10.3389/fphys.2018.00394 29740333 PMC5928447

[B106] Wal P. Tyagi S. Pal R. S. Yadav A. Jaiswal R. (2023). A strategic investigation on diabetic nephropathy; its conceptual model and clinical manifestations: a review. Curr. Diabetes Reviews 19 (5), e260422204036. 10.2174/1573399818666220426091238 35993472

[B107] Wang M. Hu R. Wang Y. Liu L. You H. Zhang J. (2019). Atractylenolide III attenuates muscle wasting in chronic kidney disease via the oxidative stress-mediated PI3K/AKT/mTOR pathway. Oxidative Medicine Cellular Longevity 2019, 1875471. 10.1155/2019/1875471 31178951 PMC6501186

[B108] Wang L. Feng S. Luo W. Xie M. Liu J. (2020). Effect of Yupingfeng Granule combined with tacrolimus on renal function, immune function and Th1/Th2 cell balance in children with primary nephrotic syndrome. Prog. Mod. Biomed. 20 (17), 3354–3357+3340. 10.13241/j.cnki.pmb.2020.17.035

[B109] Wang H. Liu D. Zheng B. Yang Y. Qiao Y. Li S. (2023). Emerging role of ferroptosis in diabetic kidney disease: molecular mechanisms and therapeutic opportunities. Int. Journal Biological Sciences 19(9)**,** 2678–2694. 10.7150/ijbs.81892 PMC10266077 37324941

[B110] Wang Q. Li J. Chen L. (2025). Traditional Chinese medicine phytochemicals: promising therapeutic agents for acute kidney injury. Integr. Med. Nephrol. Androl. 12 (4), e25–e37. 10.1097/IMNA-D-25-00037

[B111] Wei X. Gao P. Pu Y. Li Q. Yang T. Zhang H. (2017). Activation of TRPV4 by dietary apigenin antagonizes renal fibrosis in deoxycorticosterone acetate (DOCA)-salt-induced hypertension. Clin. Science Lond. Engl. 131 (7), 567–581. 10.1042/CS20160780 28143892

[B112] Wu R. Zhao N. Li H. Zhang W. Hua Y. Ji P. (2025). Protective effects of jiawei yupingfeng polysaccharides on cyclophosphamide-induced intestinal injury in mice. Int. Journal Biological Macromolecules 310 (Pt 3), 143405. 10.1016/j.ijbiomac.2025.143405 40274150

[B113] Xia P. Gao K. Xie J. Sun W. Shi M. Li W. (2020). Data mining-based analysis of Chinese medicinal herb formulae in chronic kidney disease treatment. Evidence-based Complementary Alternative Medicine eCAM 2020, 9719872. 10.1155/2020/9719872 32047530 PMC7003280

[B114] Xu H. Song H. (2025). Yu-Ping-Feng formula: a promising traditional Chinese medicine for the treatment of primary sjögren's syndrome. J. Leukocyte Biology 117 (2), qiae193. 10.1093/jleuko/qiae193 39215570

[B115] Xu Z. Wang X. Kuang W. Wang S. Zhao Y. (2023). Kaempferol improves acute kidney injury via inhibition of macrophage infiltration in septic mice. Biosci. Reports 43 (7), BSR20230873. 10.1042/BSR20230873 PMC10372469 37440431

[B4] Yan L. J. (2021). Folic acid-induced animal model of kidney disease. Animal Models Experimental Medicine 4 (4), 329–342. 10.1002/ame2.12194 34977484 PMC8690981

[B116] Yao F. Yuan Q. Song X. Zhou L. Liang G. Jiang G. (2020). Yupingfeng granule improves Th2-Biased immune state in microenvironment of hepatocellular carcinoma through TSLP-DC-OX40L pathway. Evidence-based Complementary Alternative Medicine eCAM 2020, 1263053. 10.1155/2020/1263053 32351590 PMC7171663

[B117] Yao M. Qin S. Xiong J. Xin W. Guan X. Gong S. (2022). Oroxylin A ameliorates AKI-to-CKD transition through maintaining PPARα-BNIP3 signaling-mediated mitochondrial homeostasis. Front. Pharmacology 13, 935937. 10.3389/fphar.2022.935937 PMC9445212 36081929

[B118] Yeh T. H. Tu K. C. Wang H. Y. Chen J. Y. (2024). From acute to chronic: unraveling the pathophysiological mechanisms of the progression from acute kidney injury to acute kidney disease to chronic kidney disease. Int. Journal Molecular Sciences 25 (3), 1755. 10.3390/ijms25031755 38339031 PMC10855633

[B119] Yin F. Lan R. Wu Z. Wang Z. Wu H. Li Z. (2019). Yupingfeng polysaccharides enhances growth performance in qingyuan partridge chicken by up-regulating the mRNA expression of SGLT1, GLUT2 and GLUT5. Veterinary Medicine Science 5 (3), 451–461. 10.1002/vms3.167 30973212 PMC6682804

[B120] Yuan Q. Tang B. Zhang C. (2022). Signaling pathways of chronic kidney diseases, implications for therapeutics. Signal Transduction Targeted Therapy 7 (1), 182. 10.1038/s41392-022-01036-5 35680856 PMC9184651

[B121] Zaidi S. N. F. Usman S. M. (2021). Salicylic acid attenuates gentamicin-induced nephrotoxicity in rabbits. Pak. Journal Pharmaceutical Sciences 34 (1), 165–170. 34248016

[B122] Zhang L. R. Tang Y. Jiang G. R. (2012). The protection of yupingfeng powder on cisplatin induced oxidative damage of organs in hepatocellular carcinoma mice. Chin. J. Integr. Traditional West. Med. 32 (5), 647–651. 22679727

[B123] Zhang L. Shergis J. L. Yang L. Zhang A. L. Guo X. Zhang L. (2019). Astragalus membranaceus (Huang Qi) as adjunctive therapy for diabetic kidney disease: an updated systematic review and meta-analysis. Ournal Ethnopharmacology 239, 111921. 10.1016/j.jep.2019.111921 31034954

[B124] Zhang L. Wang X. Wang D. Guo Y. Zhou X. Yu H. (2022). Adjuvant treatment with yupingfeng granules for recurrent respiratory tract infections in children: a systematic review and meta-analysis. Front. Pediatrics 10, 1005745. 10.3389/fped.2022.1005745 PMC9811950 36619520

[B125] Zhang N. Guan C. Liu Z. Li C. Yang C. Xu L. (2022). Calycosin attenuates renal ischemia/reperfusion injury by suppressing NF-κB mediated inflammation via PPARγ/EGR1 pathway. Front. Pharmacology 13, 970616. 10.3389/fphar.2022.970616 PMC9585199 36278223

[B126] Zhang N. X. Guan C. Li C. Y. Xu L. Y. Xin Y. L. Song Z. (2024). Formononetin alleviates ischemic acute kidney injury by regulating macrophage polarization through KLF6/STAT3 pathway. Am. Journal Chin. Medicine 52 (5), 1487–1505. 10.1142/S0192415X24500587 39169449

[B127] Zhang T. Widdop R. E. Ricardo S. D. (2024). Transition from acute kidney injury to chronic kidney disease: mechanisms, models, and biomarkers. Am. Journal Physiology. Ren. Physiology 327 (5), F788–F805. 10.1152/ajprenal.00184.2024 39298548

[B128] Zhang X. Wang J. Xiang S. Zhao L. Lv M. Duan Y. (2024). Astragaloside I from Astragalus attenuates diabetic kidney disease by regulating HDAC3/Klotho/TGF-β1 loop. Am. Journal Chin. Medicine 52 (6), 1795–1817. 10.1142/S0192415X24500708 39347955

[B129] Zhao J. Guo L. (2009). Research progress on the treatment of kidney diseases with Yipingfeng powder. Guid. J. Traditional Chin. Med. Pharm. 15 (9), 72–74. 10.3969/j.issn.1672-951X.2009.09.043

[B130] Zhao Y. Wang P. Sang S. (2019). Dietary genistein inhibits methylglyoxal-induced advanced glycation end product formation in mice fed a high-fat diet. J. Nutrition 149(5)**,** 776–787. 10.1093/jn/nxz017 31050753

[B131] Zhou C. J. Ma F. Liao W. J. Song L. J. Yu D. Song Y. N. (2019). Restoration of immune suppressor function of regulatory B cells collected from patients with allergic rhinitis with Chinese medical formula Yupingfeng san. Am. Journal Translational Research 11 (3), 1635–1643. 30972189 PMC6456564

[B132] Zhou N. Zhong S. Y. Gao P. He F. F. Zhang C. (2026). Hypertension-induced renal injury: from pathophysiology to therapeutic perspectives. Biomedicines 14 (3), 595. 10.3390/biomedicines14030595 41898242 PMC13024177

[B133] Zhu B. Ni Y. Gong Y. Kang X. Guo H. Liu X. (2023). Formononetin ameliorates ferroptosis-associated fibrosis in renal tubular epithelial cells and in mice with chronic kidney disease by suppressing the Smad3/ATF3/SLC7A11 signaling. Life Sciences 315, 121331. 10.1016/j.lfs.2022.121331 36586573

[B134] Zhu H. He L. Wu W. Duan H. Chen J. Xiao Q. (2023). A compounds annotation strategy using targeted molecular networking for offline two-dimensional liquid chromatography-mass spectrometry analysis: Yupingfeng as a case study. J. Chromatography. A 1702, 464045. 10.1016/j.chroma.2023.464045 37236139

[B135] Zhu Y. Lv C. Qiao Y. Yang H. Lin W. Wang X. (2025). Transformation of acute kidney injury to chronic kidney disease: the interaction between mitophagy and NLRP3 inflammasome. Front. Mol. Biosci. 12, 1643829. 10.3389/fmolb.2025.1643829 41069682 PMC12504097

[B27] Zou Y. Yu Y. T. Zang Z. L. (2015). Clinical observation of chronic glomerulonephritis treated with YupingFeng San. World Latest Med. Inf. 15 (A2), 38+40. 10.3969/j.issn.1671-3141.2015.102.024

